# CRL4 regulates recombination and synaptonemal complex aggregation in the *Caenorhabditis elegans* germline

**DOI:** 10.1371/journal.pgen.1008486

**Published:** 2019-11-18

**Authors:** Benjamin Alleva, Sean Clausen, Emily Koury, Adam Hefel, Sarit Smolikove

**Affiliations:** The department of Biology, The University of Iowa, Iowa City, Iowa, United States of America; Northwestern University, UNITED STATES

## Abstract

To maintain the integrity of the genome, meiotic DNA double strand breaks (DSBs) need to form by the meiosis-specific nuclease Spo11 and be repaired by homologous recombination. One class of products formed by recombination are crossovers, which are required for proper chromosome segregation in the first meiotic division. The synaptonemal complex (SC) is a protein structure that connects homologous chromosomes during meiotic prophase I. The proper assembly of the SC is important for recombination, crossover formation, and the subsequent chromosome segregation. Here we identify the components of Cullin RING E3 ubiquitin ligase 4 (CRL4) that play a role in SC assembly in *Caenorhabditis elegans*. Mutants of the CRL4 complex (*cul-4*, *ddb-1*, and *gad-1*) show defects in SC assembly manifested in the formation of polycomplexes (PCs), impaired progression of meiotic recombination, and reduction in crossover numbers. PCs that are formed in *cul-4* mutants lack the mobile properties of wild type SC, but are likely not a direct target of ubiquitination. In *C*. *elegans*, SC assembly does not require recombination and there is no evidence that PC formation is regulated by recombination as well. However, in one *cul-4* mutant PC formation is dependent upon early meiotic recombination, indicating that proper assembly of the SC can be diminished by recombination in some scenarios. Lastly, our studies suggest that CUL-4 deregulation leads to transposition of the Tc3 transposable element, and defects in formation of SPO-11-mediated DSBs. Our studies highlight previously unknown functions of CRL4 in *C*. *elegans* meiosis and show that CUL-4 likely plays multiple roles in meiosis that are essential for maintaining genome integrity.

## Author summary

Defects in the formation of the structure named the synaptonemal complex (SC) lead to the missegregation of chromosomes in the divisions that generate sperm and egg cells. In humans, this chromosome missegregation is associated with infertility and developmental disabilities of the surviving progeny. Abnormal SC structures composed of misfolded and aggregated SC proteins are associated with an inability to properly repair DNA damage and accurately segregate meiotic chromosomes. How SC proteins assemble such that they do not form misfolded protein aggregates is poorly understood. The germlines of nematodes (*Caenorhabditis elegans*) that lack protein components of the Cullin 4 E3 Ubiquitin ligase complex (CRL4), have defects in the formation of the SC that can be due to misfolding of SC proteins and their aggregation. CRL4 appears to be involved in other germline functions that directly affect chromosome stability (DNA damage repair and transposition), indicating that CRL4 has a central function in the formation of functional sperm and egg cells.

## Introduction

Meiosis is a specialized cellular division essential for sexually reproducing metazoans. Meiosis proceeds by two cellular divisions: the first separates homologous chromosomes and the second separates sister chromatids. Perturbations of meiosis can lead to missegregation of chromosomes and nondisjunction resulting in aneuploidy and/or inviable offspring (reviewed in: [1,2]). Prior to the first division, during meiotic prophase I, crossovers between homologous chromosomes promote proper segregation in the subsequent division. In *Caenorhabditis elegans*, homologous chromosomes pair, synapse, and proceed through meiotic recombination (reviewed in: [3]). Synapsis in some organisms is a process that can initiate concurrently with pairing (typically in the leptotene/zygotene stages), and involves the assembly of the tripartite protein structure, the synaptonemal complex (SC) [2]. Assembly of the SC occurs between homologous chromosomes; once SC is present along the entirety of the chromosome, the chromosomes are synapsed. In sexually reproducing model organisms, DSB formation and repair require specific components of the SC that are part of the chromosome axis (reviewed in: [4]). SC assembly is not always dependent upon DSB formation and recombination initiation; some species do not require DSB formation or recombination for synapsis (*e*.*g*. *Drosophila*; [5] and *C*. *elegans*; [6]). In other cases, SC assembly depends on DSB formation/recombination (*e*.*g*. mouse; [7] and *S*. *cerevisiae*; [8]). Negative regulation of SC assembly, or prevention of aberrant SC formation, by recombination has not yet been described.

Meiotic recombination initiates by DNA double strand break (DSB) formation. Meiotic DSBs are typically formed by Spo11, a topoisomerase VI-like protein [9]. Upon DSB formation, nucleolytic excision of the 5’ DNA strand occurs, leaving a 3’ single strand DNA overhang. This overhang is subsequently bound by the RecA homolog, Rad51/Dmc1; the DNA-protein complex invades a homologous chromosome as a template for repair. Synthesis dependent repair follows ultimately resulting in the formation of non-crossovers and crossovers. On each chromosome a crossover, together with sister chromatid cohesion, physically hold homologs together. The SC then disassembles, and chromosomes await separation in metaphase I. In aberrant conditions, DSBs can occur in the germline through other mechanisms, such as movement of transposable elements (TEs) (*e*.*g*., [10,11]). To prevent this, the movement of TEs is suppressed by germline-specific RNA interference mechanisms (*e*.*g*., [12–14]).

The SC in *C*. *elegans* is composed of two main components, the axial element and the central region. The axial element is made up of four proteins: HTP-1/2, HTP-3, and HIM-3, and assembles first along each homolog [15–18]. This allows the central region to assemble, also made up of four proteins: SYP-1-4, which connect the two homologs, stabilizing pairing [19–22]. Loss or disturbance of any one of these four central components inhibits the other components’ ability to localize and assemble. Studies of the SC in *C*. *elegans* revealed its dynamic structure: even when fully assembled SC proteins still are being exchanged [23,24]. The SC is more dynamic in early and mid-prophase and its dynamic properties are more restricted once crossovers are formed in late pachytene. This communication between the crossovers to the SC was shown to be regulated by SYP-4 phosphorylation [23]. While the processes controlling SC dynamics may be related to SC assembly, so far recombination appears to play no role in SC assembly; in the absence of SPO-11, or any protein involved in recombination, the SC assembles normally as in wild type. One exception is observed in the *cra-1* mutant background in which SC assembly defects become more severe in the absence of early meiotic recombination intermediates [25]. How, and under what conditions, recombination affects SC assembly in *C*. *elegans*, is not yet understood.

**Fig 1:**
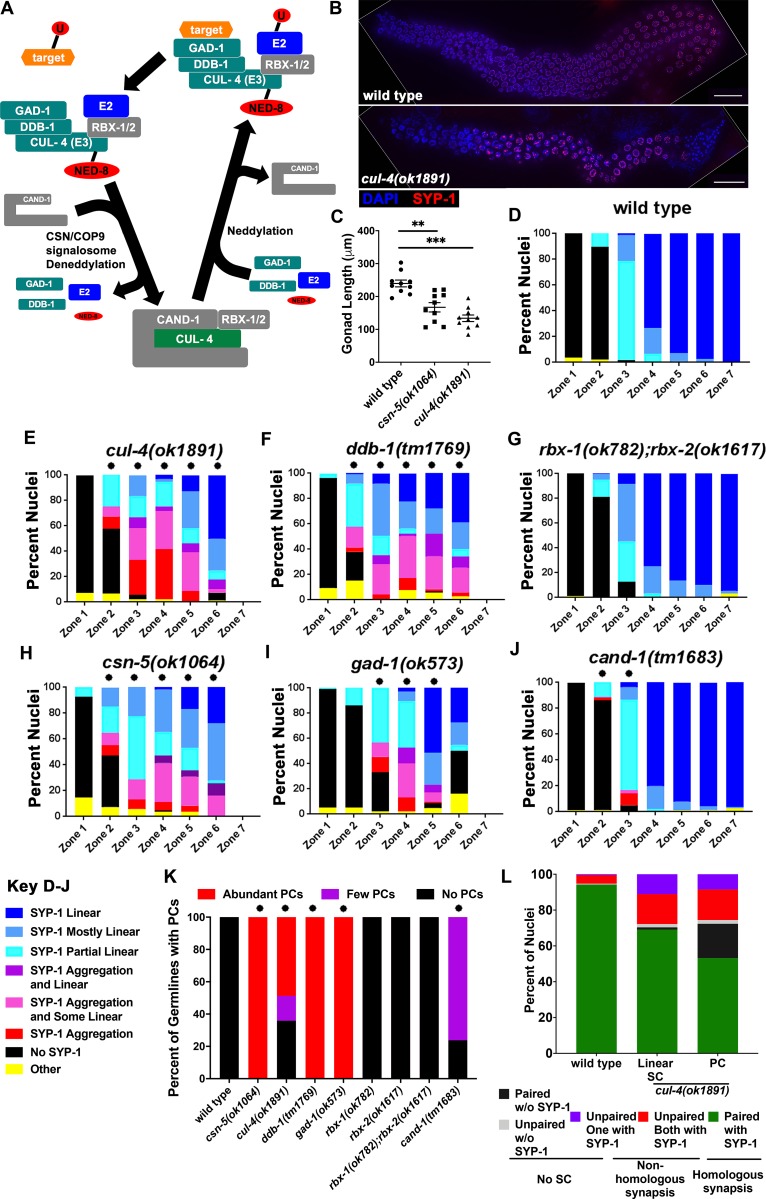
CUL-4 E3 ligase complex mutants exhibit PC formation in meiotic prophase I. A) A model of the CRL4 E3 ligase complex and its components examined in this study, based on what is known about its biochemical activity and composition from studies in other systems. The complex is presented in two states: active (neddylated/upper) and inactivated (deneddylated/lower), the active form is depicted before and after ubiquitination of the substrate. Gray subunits represent deletion mutations that do not result in a phenotype (RBX-1/2) or show minor phenotype (CAND-1) B) Full germline images of wild type (top) and *cul-4(ok1891)* mutants (bottom); blue (DAPI) and red (SYP-1). White lines are the border between the image and a black background that was added so a rectangular shape will be created. Scale bars are 20μm. C) Graphical comparison of germline length in wild type, *csn-5(ok1064)*, and *cul-4(ok1891)* mutants (Mann-Whitney; p-values, * < 0.001). D-J) Graphical analyses of SYP-1 localization analysis (“No SYP-1” was defined as having no SYP-1 immunofluorescence present along chromosomes (DAPI). “SYP-1 PC” were nuclei with only PC formation present, no elongation of SYP-1 along DAPI. “SYP-1 PC and Some Linear” was the presence of PC(s) in the nucleus but also partial elongation of SYP-1, <50% of DAPI. “SYP-1 PC and Linear” was similar to “SYP-1 PC and Some Linear” with the exception that SYP-1 is elongated along >50% of DAPI. “SYP-1 Partial Linear” nuclei had elongated SYP-1 along up to 50% of DAPI. “SYP-1 Mostly Linear” nuclei had SYP-1 along up to 50% of DAPI but less than 100%. “SYP-1 Linear” nuclei had fully elongated SYP-1 along all DAPI. “Other” was defined as nuclei that had abnormal DAPI appearance.); statistical comparisons of each mutant were made against wild type (Fischer’s exact test; p-values, * < 0.001). Scale bars are 2μm. K) Analysis of percentage of germlines with PC formation within each individual genotype. The number of PCs per germline were grouped into three main categories: No PCs = germlines with no SC PC formation, Few PCs = germlines no more than 5 PCs or less than ½ of a zone with PCs, and Abundant PCs = germlines with more than 5 PCs or greater than ½ of a zone with PCs. Statistical comparisons were made to wild type for each mutant individually (Fisher’s exact test; p-values, * < 0.0001). L) Analysis of homologous and non-homologous pairing by FISH to the 5S locus coupled with SYP-1 immunostaining in pachytene nuclei. In wild type the majority of nuclei are paired and synapsed (green) while in *cul-4(ok1891)* mutants less nuclei are paired. Unpaired FISH foci frequently associated with SYP-1 on at least one locus (purple and red) indicating non-homologous synapsis occurs in *cul-4(ok1891)* mutants regardless if the nuclei contain or do not contain PC. *cul-4(ok1891)* nuclei with PCs also contain FISH singnals that do not associate with SC (black and gray).

The post-translational modifier, ubiquitin, is known to play an important part in meiosis in several organisms (reviewed in: [26,27]). Ubiquitination involves a protein cascade; the E1 enzyme activates ubiquitin so the E2 conjugation enzyme can transfer ubiquitin to the E3 ubiquitin ligases. E3 enzymes then attach ubiquitin to target proteins (Fig 1A). The most well-known function of ubiquitination is targeted degradation by the proteasome. Loss of some E3 activity or inhibition of the proteasome in meiosis can lead to delayed meiotic entry (*C*. *elegans*, and mice; [28,29]), improper meiotic recombination (yeast, *C*. *elegans*, and mice; [30–32]), and perturbed assembly of SC central elements (*C*. *elegans*; [33,34]).

One of the major families of ubiquitin ligases are the Cullin Ring E3 ubiquitin ligases (CRL) which are activated by neddylation. The activity of CRLs are controlled by deneddylation through the CSN/Cop9 Signalosome. One particular CRL, Cullin RING E3 ubiquitin ligase 4 (CRL4), has been shown to play an important role in the processes required for proper DNA function and maintenance (*e*.*g*. DNA repair, histone deposition, and replication (reviewed in: [35]). CRL4 is typically composed of 4 subunits; a Cullin scaffold protein, a RING box protein (Rbx) that binds the E2 enzyme, an adaptor protein that binds the ubiquitin-targeted substrate, and Ddb1 which is a mediator between the cullin scaffold and the adaptor protein. While Cul4, Ddb1, and Rbx are found in all CRL4s, the adaptor protein varies and is specific to a few substrates (reviewed in: [36]). In *C*. *elegans*, one known role of CRL4 is in the prevention of re-replication during S-phase in the soma [37–39], but not in the germline [40]. In meiosis, deletion of Cul4A in mice leads to defects in recombination and increased apoptosis [29,41], and in Arabidopsis Cul4 mutants affect the distribution of crossovers [42]. We previously identified CUL-4 as a member of the SC assembly pathway in *C*. *elegans* meiosis, and CRL4 is a plausible target of the CSN/Cop9 signalosome [34]. In wild type *C*. *elegans*, recombination depends on SC assembly but not vice versa [6,22]. Therefore, we suggested that the defects in recombination identified in this mutant are due to SC assembly defects [34]. The role of other components of CRL4 on SC assembly were unknown, nor the identity of the adaptor protein. In this study, we determined novel roles for the CUL-4 E3 ubiquitin ligase complex in meiotic prophase I by examining oogenesis in the model organism *C*. *elegans*.

We examined four proteins (CUL-4, DDB-1, GAD-1, and RBXs) that were part of the CRL4 E3 ligase complex to determine if they were involved with SC assembly and/or meiotic recombination. We show that in *C*. *elegans*, the CRL4 E3 ligase plays a role in meiotic homologous recombination. Surprisingly, SC proteins are affected by meiotic recombination in one *cul-4* mutant, as inactivation of proteins involved in meiotic recombination prevents PC formation in this *cul-4* mutant. CUL-4 also appears to have a role in some TE silencing leading to another source of DNA damage. Our studies suggest that CUL-4 performs a conserved role in meiosis in DSB repair and reveal a novel effect of recombination on SC assembly behavior.

## Results

### SC assembly is perturbed in CRL4 mutants

We have shown previously that the CUL-4 scaffold of the CRL4 ubiquitin ligase is required for proper SC assembly in *C*. *elegans* [34]. The CUL-4 RING E3 Ligase (CRL4) complex is composed of 4 subunits, some of which vary between organisms and processes [35]. In *C*. *elegans*, the CRL4 complex is made up of four main components: CUL-4, the main scaffolding protein for the complex, a RING box protein that interacts with the E2 conjugating enzyme for targeted ubiquitination, DDB-1 which binds CUL-4 so that an adaptor protein can bind to the complex, and an adaptor protein (DDB1-Cul4-associated factor, DCAF) that recognizes target proteins for ubiquitination (Fig 1A). We aimed to identify proteins that were part of the CRL4-mediated, SC assembly pathway by focusing on candidates that were part of the CRL4 in other cellular functions, as well as through a targeted RNAi screen (described in Materials and Methods). CUL4, DDB1 and RBX homologs have been previously found in *C*. *elegans*. CUL-4 and DDB-1 were shown to have a conserved role in mitotic replication and physically interact with each other[40,43]. RBX-1 acts with other CRL complexes found in *C*. *elegans*, but has not been shown to act within CRL4[44]. To identify the adaptor protein we performed an RNAi screen of genes encoding for proteins containing a WD40-DDB1 domain (see below).

To examine SC assembly dynamics, immunofluorescent staining was performed on dissected *C*. *elegans* germlines (Fig 1B and S1 Fig). Nuclei in the germlines are positioned in a sequential manner allowing for a time course analysis of early meiotic events. Based on the timing of HTP-3 and SYP-1 localization we can conclude that meiotic entry occurs in the genotypes tested (S1K-N and [34]). To properly compare dynamics of SC assembly, length measurements of wild type, *csn-5(ok1064)*, and *cul-4(ok1891)* mutant germlines were taken. Both *cul-4(ok1891)* and *csn-5(ok1064)* germlines were significantly shorter than wild type germlines (*cul-4(ok1891)*: p<0.0001, *csn-5(ok1064)*: p = 0.0007, Mann-Whitney, Fig 1C). These analyses were used as a basis to define the progression of meiotic prophase I, in that each zone, or image, represents a different portion of the pachytene time-course (description in Materials and Methods). In wild type germlines, Zones 1 and 2 correspond to the pre-meiotic tip (PMT) of the germline where mitotic proliferation occurs prior to meiotic entrance. In *cul-4(ok1891)* and *csn-5(ok1064)* mutants, as well as other CRL4 mutants, Zone 1 and the initial portion (approximately 1/3) of Zone 2 represent the PMT. In wild type, Zones 3–7 progressively represent meiotic prophase I. Zone 3 includes leptotene/zygotene, where SC assembly initiates and SPO-11 dependent DNA double strand breaks (DSBs) occur; Zones 4–7 represent pachytene, where SC assembly is completed and meiotic recombination is finished. Based on the timing of HTP-3 and SYP-1 localization to chromosomes in *cul-4(ok1891)* and *csn-5(ok1064)* mutants (Fig 1D, 1E and 1H; S1K and S1L Fig), the latter portion of Zone 2 and early Zone 3 are leptotene/zygotene, the rest of Zone 3 and Zones 4–6 are pachytene. The difference in zones between genotypes (e.g. wild type vs. *cul-4(ok1891)* mutants) was due to a reduction in the size of the mitotic proliferation zone (Fig 1D, 1E and 1H; S1K and S1L Fig and [34]) and is consistent with the reduced gonad size (Fig 1C).

SC assembly can be observed by staining with SYP-1, a component of the central region of the SC. In wild type, SYP-1 is absent in PMT nuclei. Upon meiotic entrance, SYP-1 begins to assemble (in a linear pattern) along homologous chromosomes and becomes fully assembled along homologs in early pachytene (Fig 1D; S1A Fig). In *cul-4(ok1891)* mutants, large polycomplexes (PCs) are formed, which are defined as at least twice the width of typical SYP-1 structures [34] found in wild type nuclei [Fig 1D *vs*. 1E; S1B Fig; PCs (purple, pink and red categories) are absent in wild type but present in ~half of meiotic prophase I nuclei of *cul-4(ok1891)* mutants]. These PCs may be due to SC assembly defects suggesting a role for CRL4 in SC assembly, consistent with [34]. Although PCs were abundant in *cul-4(ok1891)* mutants, nuclei frequently showed linear SC even in the presence of PCs. It is possible that this SC would not form between homologs, leading to non-homologous synapsis. Analysis of SYP-1 combined with FISH showed that most SC assembled between homologs (Fig 1L). However, about 25% of *cul-4(ok1891)* mutant nuclei showed non-homologous synapsis of at least one chromosome, regardless whether they exhibit PCs or only linear SC (Fig 1L).

Only 64% (n = 23/36) of *cul-4(ok1891)* mutant germlines have PCs (Fig 1K). This incomplete penetrance of *cul-4(ok1891)* is observed with a 6X outcrossed line and is independent of the balancer used, indicating that the incomplete penetrance phenotype is specific to *cul-4(ok1891)*. We monitored the incomplete penetrance another way to determine if the phenotype could be observed in a single worm or if the phenotype differed between individuals. Using a *gfp*::*syp-3* construct, SC assembly was monitored in the two gonadal arms of each worm allowing for a thorough analysis of incomplete penetrance. When *gfp*::*syp-3* was present in a *cul-4(ok1891)* mutant background, 49% of worms (n = 33, S1C Fig) had only one arm with PCs. This supports our view that the phenotype is due to the *cul-4(ok1891)* allele since the genetic background of the two arms is, of course, identical. *cul-4* null mutant strains containing the *gk434* or *gk511* alleles or *cul-4(RNAi)* treatment arrest at the L2 larval stage and do not have germlines that can be scored ([38]and this study). In agreement, *gk434* and *gk511* alleles are expected to lead to larger deletion of the protein compared to *ok1891*. RT-PCR of *cul-4(ok1891)* revealed that this mutant has an out-of-frame deletion with a premature stop codon in the *cul-4* transcript. *cul-4(ok1891)* is expected to encode for a C’ truncated protein that does not contain both the RBX/ROC binding site and the neddylation site of CUL-4. Thus, *cul-4(ok1891)* likely exhibits partial penetrance as it is not a full loss-of-function allele.

The CRL4 complex involved in prevention of re-replication is composed of RBX-1 [45] and DDB-1 [40], which are also predicted to be obligatory CRL4 subunits in other species [35]. We hypothesized that if RBX-1 and DDB-1 interact with CUL-4, in meiosis, then *ddb-1(tm1769)* and *rbx-1(ok782)* mutants should exhibit a phenotype similar to that of *cul-4(ok1891)*. As expected, *ddb-1(tm1769)* and *cul-4(ok1891)* mutant germlines had PC formation with similar distributions throughout the germline (Fig 1F; S1D Fig). Unlike *cul-4(ok1891)* mutants, *ddb-1(tm1769)* showed 100% penetrance, meaning all germlines analyzed had PCs (Fig 1K). Complete penetrance is consistent with these alleles behaving as null, as predicted from the size and positions of the deletions in these alleles.

Next, we examined the phenotype of mutations in the two *rbx* genes of *C*. *elegans*. *rbx-1(ok782)* is a deletion of the entire gene, while *rbx-2(ok1617)* removes the last 48% of the coding sequence. Interestingly, neither *rbx-1(ok782)* nor *rbx-2(ok1617)* mutants had defects in SC assembly (S1E & S1F Fig). To test for a possible redundant function, *rbx-1(ok782);rbx-2(ok1617)* double mutants were created; these mutants also showed no sign of PC formation (Fig 1G; S1G Fig). This suggests that a RING box protein may not be required for SC assembly or that a non-canonical RING protein is involved in this function (Fig 1A and Discussion). These data collectively show that the CRL4 (CUL4 and DDB-1), as well as its regulation (CSN-5: Fig 1H; S1H Fig; [34]) are important for proper SC assembly, independent of RBX-1/2.

To identify the adaptor component of the CRL4 that is involved in SC assembly, we conducted a targeted screen using candidate genes that encode for proteins with WD40-DDB1 interaction domains (see Materials and Methods). This screen led to the isolation of *gad-1* as a candidate gene. The *gad-1(ok573)* mutant exhibited PC formation, similar to *ddb-1(tm1769)* (Fig 1I; S1I Fig). Like *ddb-1(tm1769)* mutants, *gad-1(ok573)* showed 100% penetrance, all germlines analyzed had PCs (Fig 1K). We therefore propose that the CRL4 complex that is required for proper SC assembly includes DDB-1 and GAD-1 but not RBX-1 or RBX-2.

We have previously shown that *csn-5(ok1064)* mutants exhibited PC formation throughout meiotic prophase I [34]. This suggests that overactive (neddylated) CRL4s that cannot be recycled lead to PC formation (Fig 1H; S1H Fig). CAND-1 is a known regulator of cullin neddylation through its ability to prevent Cullin-RING E3 ligase neddylation [46]. However, unlike other proteins in this pathway, *cand-1* is not an essential gene (*cand-1(tm1683)* homozygote mutants are viable [46]). CAND-1 is proposed to sequester inactive CRL4 following its deneddylation, prolonging its time in the deactivated form. If the balance between neddylated and deneddylated forms is important for proper CRL4 activity in SC assembly, then inactivation of *cand-1* should result in defects in SC assembly. We observed that *cand-1(tm1683)* mutants had a small percentage of nuclei with PCs, observed only in Zones 2 and 3 (Fig 1J and 1K; S1J Fig). This indicates that the sequestering of inactive CRL4s is not important for SC assembly as there are very few PCs formed in *cand-1(tm1683)* mutants.

To determine where the CRL4 complex is localized, we tagged three of the CRL4 complex proteins by CRISPR/Cas9 insertion of sequence tags encoding for OLLAS and FLAG. CUL-4, GAD-1, and DDB-1 were all enriched in germline nuclei, consistent with their proposed meiotic function (S2 Fig).

### Depletion of CUL-4 levels induces PC formation

**Fig 2:**
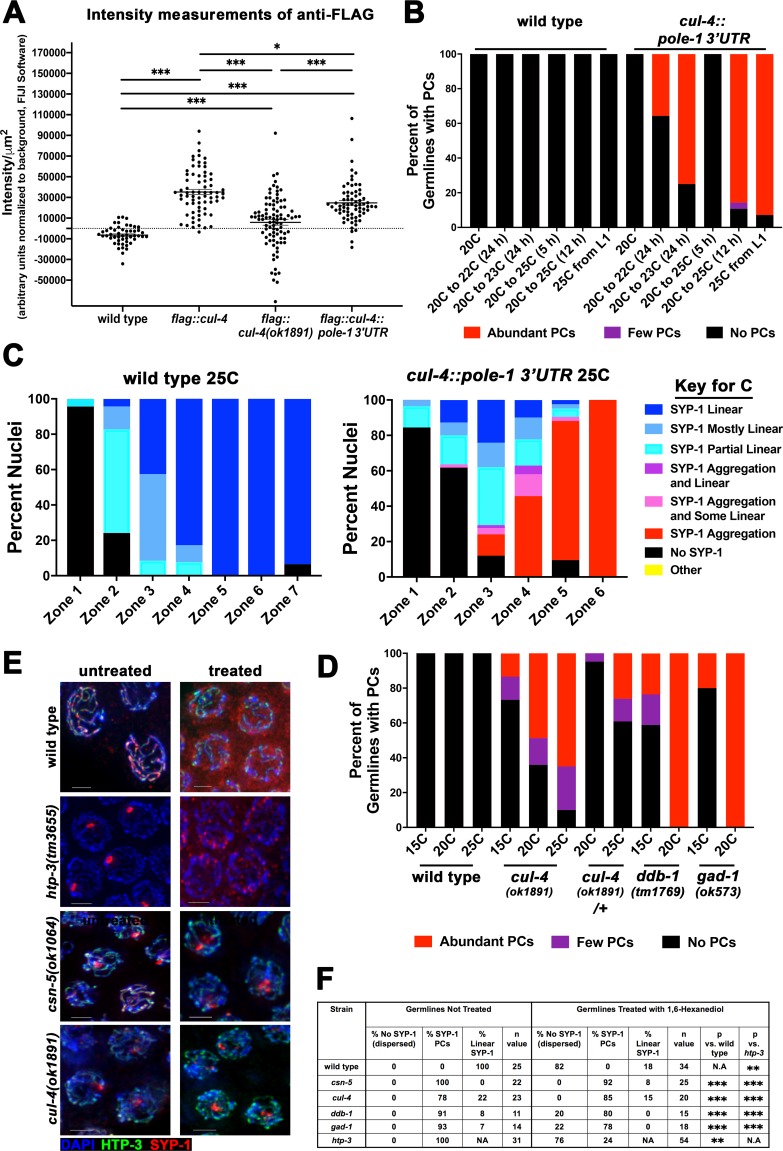
PCs are formed in CRL mutants due to protein misfolding. A) FLAG::CUL-4 localization was quantified in LP nuclei in wild type nuclei, and CUL-4 tagged lines: *flag*::*cul-4*, *flag*::*cul-4(ok1891)*, and *flag*::*cul-4*::*pole-1 3’UTR*. Cytoplasmic background was subtracted from wild type staining (no FLAG), leading to negative average values for the untagged wild type strain, while all other genotypes had positive average values, indicating nuclear staining. Intensity is in arbitrary units. B) *cul-4* mutant with 3’UTR replacement shows temperature dependent PC formation. Categorization of germlines indicated below the graph. The categories are presented next to the graph (Abundant PC in red, Few PC in purple, No PC in black). Temperatures are presented on the X axis. For example, “20C to 25C(12h)” means: worms were grown from egg at 20C, and shifted at the last 12 hours before analysis to 25C. All worms analyzed as 1 day old adults. C) Whole germline analysis of SC morphology in *cul-4* mutants with 3’UTR replacement grown at 25C from the L1 stage. Wild-type worms were grown under the same conditions and exhibit no PC formation. Categories on the right. D) CRL4 mutant with 3’UTR replacement shows temperature dependent PC formation. Categorization of germlines indicated below the graph. E) Representative images of untreated and 1,6-Hexanediol treated germlines in wild type, *htp-3(tm3655)*, *csn-5(ok1064)*, and *cul-4(ok1891)* strains. Blue (DAPI), green (HTP-3), and red (SYP-1). Images were taken from mid-pachytene (Zone 4/5). Scale bars are 2μm. F) Results of 1,6-Hexanediol treated germlines. Categorization of SYP-1 immunofluorescent staining after 1,6-Hexanediol treatment are defined as follows: “No SYP-1 (dispersed)” represents germlines where nuclei no longer have SYP-1 localization to chromosomes, “PCs” represents germlines where nuclei have PCs localized to chromosomes (for non-treated these also contain linear SC alongside PCs, and treated did not contain linear SC), and “Linear SYP-1” represents germlines where nuclei have linearized SYP-1 localized to chromosomes. All germlines had linear HTP-3 staining. Statistical comparisons were performed versus wild type worms (Fisher’s exact test dispersed or with SYP-1 vs. PC; p-values, *** p< 0.0001, ** 0.01> p> 0.001).

The *cul-4(ok1891)* mutant is not a null allele as these worms are able to develop to adulthood, whereas successful RNAi of *cul-4* and other mutants of the gene (null alleles) result in larval lethality. Thus, the *cul-4(ok1891)* mutant phenotype can be attributed either to a reduction in CUL-4 protein levels or to the formation of a truncated CUL-4 protein. To test if *cul-4(ok1891)* mutants have reduced levels of CUL-4 protein, we introduced a FLAG tag to the *cul-4(ok1891)* mutants and quantified the nuclear localization of CUL-4 by FLAG staining intensity (Fig 2A; S2C Fig). As expected, CUL-4 was present in *cul-4(ok1891)* mutants but at reduced levels in late pachytene nuclei (~15% of wild type) indicating reduced CRL4 levels. Interestingly, the variation in nuclear localization of CUL-4 in *cul-4(ok1891)* mutants was high and 25% of nuclei had no detectable levels of FLAG::CUL-4 similar to wild type (no FLAG::CUL-4). This variability of CRL4 nuclear localization could explain the incomplete penetrance of PC formation in the *cul-4(ok1891)* mutants at 20°C (Fig 1K). However, these data do not exclude the possibility that the phenotype observed in *cul-4(ok1891)* mutants may be attributed to the formation of aberrant CRL4 complexes in these mutants (see below).

If the phenotype of *cul-4(ok1891)* mutants is caused by a reduction of CUL-4 levels, PC formation may be induced by reduction of CUL-4 levels through a different experimental approach. To do so, we mutated *cul-4* such that expression of *cul-4* was in the PMT (mitotically dividing nuclei) but not pachytene (meiotic nuclei). Expression of genes in the germline are regulated post-transcriptionally by proteins and small RNAs that bind the 3’UTR of mRNAs [47]. We took advantage of this system of regulation through CRISPR/Cas9 replacement of the 3’UTR of the endogenous *cul-4* gene with that of the 3’UTR of the polymerase epsilon gene, a replicative polymerase. This mutant should then have CUL-4 expression in the embryo, during development, and in the mitotic germline, but not in the meiotic germline. Any meiotic CUL-4 proteins present in these nuclei should be protein carried over from mitosis. We also FLAG tagged this mutant protein and observed that CUL-4 levels in the germline are reduced to ~70% of that in the wild type *flag*::*cul-4* strain, in both mitotic and meiotic nuclei (Fig 2A; S2C Fig). The fact that FLAG::CUL-4 is observed in late pachytene nuclei of *cul-4*::*pole-1 3’UTR* mutants (S2C Fig) indicates that most of the CUL-4 protein that is made in mitotic cells is not degraded upon entrance of, or in, meiotic prophase I.

Since *cul-4*::*pole-13’UTR* mutants had reduced levels of nuclear FLAG::CUL-4, we performed SYP-1 staining to test for the formation of PCs in these mutants. Unlike *cul-4(ok1891)* mutants, CRL4 that is formed in this mutant is expected to be wild type in nature (intact CUL-4 and interacts properly with other CRL4 components). *cul-4*::*pole-13’UTR* mutants did not show any PC formation at 20°C, but when worms were shifted to higher temperatures, PCs were observed (Fig 2B and 2C). As little as a 2°C temperature shift (22°C) for 24 hours could induce PC formation. Increased shifts in temperature and length of temperature exposure corresponded to an increase in number of germlines that exhibited PC formation. No PCs were observed in wild type gonads under any of the temperature shifted conditions tested. As the *cul-4(ok1891)* allele has been described before [32], we continued our studies with the *ok1891* allele as opposed to *cul-4*::*pole-13’UTR* mutants.

Based on fluorescence analysis, the level of CUL-4 protein in the *cul-4(ok1891)* mutants was lower than that in *cul-4*::*pole-13’UTR* mutants, which may explain why *cul-4*::*pole-13’UTR* mutants exhibited a phenotype at 25°C but not at 20°C. We would therefore expect that the penetrance of *cul-4(ok1891)* mutant phenotypes will be enhanced at 25°C and suppressed at 15°C. Indeed, *cul-4(ok1891)* mutants were almost completely penetrant for PC formation at 25°C and show lower penetrance at 15°C compared to 20°C (Fig 2D). If *cul-4(ok1891)* mutants exhibited defects in SC assembly due to a gain-of-function activity, we expected the CUL-4 protein generated in this mutant to be dominant. However, *cul-4(ok1891)* mutant heterozygotes [*cul-4(ok1891)*/+] show very limited PC formation that is only significant at 25°C (Fig 2D). Since *cul-4(ok1891)*/+ likely produce less CUL-4 protein product than +/+ (wild type), the appearance of PCs at 25°C can be attributed to the reduction in protein levels. These data indicate that PCs can be formed due to a reduction in CUL-4 protein levels, and not only through perturbations of CUL-4 protein function such as the gain of function activity of the *cul-4(ok1891)* allele.

The correlation between temperature and PC formation may be specific to the perturbation of CUL-4 activity, and not the CRL4 complex. To examine if other CRL4 mutants exhibit temperature-dependent PC formation as well, we have grown *ddb-1(tm1769)* and *gad-1(ok573)* mutants at 15°C. These two mutants were completely penetrant at 20°C, but had decreased penetrance of PC formation when grown at 15°C from L4 to the adult developmental stage. These data altogether indicate that perturbation of CRL4 activity correlates with defects in PC formation in a temperature-dependent manner.

### PC formation in CRL mutants are resistant to 1,6-Hexanediol treatment

PCs can be categorized into two main forms: dissolvable PCs that contain organized SC-like structures with liquid crystal properties and indissoluble PCs that have lost their liquid-like properties (Rog *et al*, 2017). The fact that the penetrance of PC formation phenotype is enhanced by increased temperature suggests that the PCs found in CRL4 mutants may be due to SYP protein misfolding, which would lead to loss of the liquid-like properties of the PCs in these mutants. There is evidence that misfolded PCs cannot be disassembled with 1,6-Hexanediol, while properly assembled SC can [48]. When treated with 1,6-Hexanediol, normal SC found in wild type germlines become mostly dispersed, no longer forming linear structures along chromosomes (82% of germlines; Fig 2E & 2F). HTP-3 is a component of the SC axial element; *htp-3(tm3655)* mutants and loss of this protein in meiotic prophase causes the formation of PCs that are known to maintain liquid-like properties (similar to linear SC). In *htp-3(tm3655)* mutants, nuclei also had mostly dispersed localization of SYP-1 (76% of germlines; Fig 2E & 2E; [48]). In both *csn-5(ok1064)* and *cul-4(ok1891)* mutants, PCs remained largely intact; only 8% and 15% of germlines had disassembled PCs, respectively (Fig 2E & 2F). Similar resistance to 1,6-Hexanediol treatment was observed in *ddb-1(tm1769)* and *gad-1(ok573)* mutants (Fig 2F) (Fig 2F). This indicates that SC assembly is perturbed in CRL4 mutants in a way that prevents dissolution of PCs, likely due to the loss of the liquid crystal properties in these PCs.

### Central region SC proteins are likely not targeted for ubiquitination by the CRL4 complex

PC formation in CRL4 pathway mutants affects all central region proteins of the SC (SYP-1/2/3/4), whereas it appears to have no effect on the axis (HTP-3). One possible explanation for PC formation in CRL4 pathway mutants is that ubiquitination of any or all SC central region components is different compared to wild type. In this model, the CRL4 E3 ligase complex would target one or more of the SYPs for ubiquitination. To examine this, we performed western blot analysis of central region SC components in wild type compared to *csn-5(ok1064)* and *cul-4(ok1891)* mutants. Ubiquitination is expected to add ~9kDa to the final protein product (or more for poly-ubiquitination). If one of the SYP proteins was ubiquitinated it should show a lower mobility shift in *cul-4(ok1891)* mutants (which lack CRL4-mediated ubiquitination) as well as a change of protein size in *csn-5(ok1064)* mutants (depending on whether these mutants contain hyper active or destabilized CRL4). To test this, we epitope tagged all four SYPs using CRISPR/Cas9 (see Materials and Methods). The observed SYP-2 molecular weight from western blot analysis was as expected (~24 kDa), while all other SYPs run at a higher molecular weight than expected (expected: 57, 26, and 67kDa, observed: ~75, ~30, and ~80kDa for SYP-1, SYP-3 and SYP-4 respectively), which may be evidence of post-translational modifications. However, examination of SYP-1 and tagged versions of SYP-2, SYP-3, and SYP-4 in both wild type and, *csn-5(ok1064)* and *cul-4(ok1891)* mutants showed no evidence for ubiquitination of any SYP protein, as the molecular weight did not change between mutants and wild-type (V5 antibody: SYP-2::V5 and V5::SYP-4, and FLAG antibody: SYP-1::FLAG and SYP-3::FLAG; S3 Fig). SYP proteins that are modified by ubiquitination may be subjected to rapid degradation. In this case, ubiquitination by CRL4 may not be detected by a standard western blot. Exposure of worms to MG132, a proteasome inhibitor, should lead to accumulation of rapidly degrading forms of SYP proteins, if they are present. However, we did not observe any change in mobility of SYP proteins under these conditions and the bands corresponding to tagged proteins showed similar mobility between wild type and *cul-4(ok1891)* mutants. In most cases, SYP levels did go down in the *cul-4* mutant, but this can be attributed to the reduction in germline size. These data altogether suggest that the effect on PC formation is likely not due to ubiquitination of the four known SYP SC proteins by the CUL-4 E3 ligase.

### CRL4 mutants show reduced pairing of homologous chromosomes

**Fig 3:**
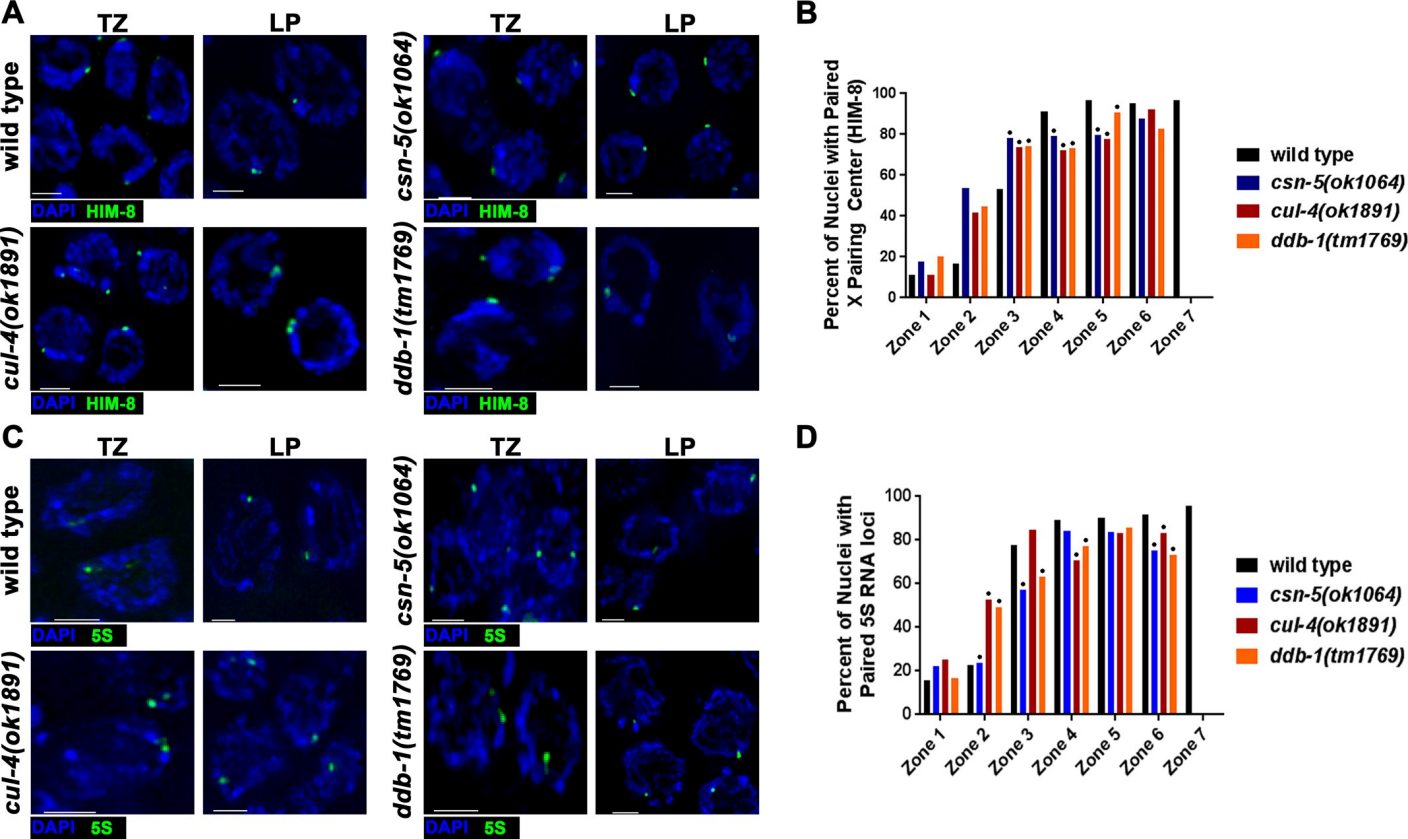
Homologous chromosome pairing levels are decreased in CRL4 E3 Ligase mutants. A) Representative images of HIM-8 (X chromosome pairing center protein) immunofluorescent antibody staining in wild type, *csn-5(ok1064)*, *cul-4(ok1891)*, and *ddb-1(tm1769)* strains. Images are taken from transition zone (TZ, Zone 2/3) and late pachytene (LP, Zone 6/7). Blue (DAPI) and green (HIM-8). B) Analysis of pairing progression in meiotic germlines. HIM-8 foci were defined as being paired if ≤ 0.7μm apart. Statistical analyses were performed in comparison to wild type worms (Fisher’s exact test; p-values, * < 0.005). C) Representative images of FISH (5S rDNA loci) in wild type, *csn-5(ok1064)*, *cul-4(ok1891)*, and *ddb-1(tm1769)* strains. Blue (DAPI) and green (5S rDNA). D) Analysis of 5S rDNA loci pairing progression per nucleus in the meiotic germline. 5S rDNA foci were defined as being paired if ≤ 0.7μm apart. Statistical comparisons were performed in comparison to wild type worms (Fisher’s exact test; p-values, * < 0.05). Scale bars are 2μm.

SC defects and PC formation often accompany a defect in homolog pairing [18,49,50] or PC formation may be due to an effect on SC assembly downstream from initiation of pairing interactions (e.g. [25,34]). To determine if homologous chromosome pairing is perturbed in CRL4 mutants, HIM-8 immunofluorescent analysis and fluorescent *in situ* hybridization (FISH) were utilized (Fig 3). HIM-8 is a pairing center protein that localizes to the X-chromosome pairing center (a small repeat sequence near the telomere) [51]. It is required for X chromosome associations with the nuclear envelope that are important for timely and proper pairing of the X chromosome. In wild type, approximately 53% of nuclei have paired X-chromosomes upon entrance into meiosis (Zone 3), and by pachytene (Zones 4–5) over 90% of nuclei have paired X homologs (Fig 3A & 3B). *cul-4(ok1891)* and *ddb-1(tm1769)*, follow a similar pattern with approximately 50% paired X-chromosomes in Zone 2, but only reach approximately 70% of paired X-chromosomes in nuclei by pachytene (Zone 3; Fig 3A & 3B). This is similar to what was previously shown for *csn-5(ok1064)* mutants ([34] and this study). To examine whether autosomal pairing is affected, FISH was performed at the 5S RNA locus on chromosome V. In wild type, approximately 80% of nuclei had paired 5S loci (Zone 3, Fig 3C & 3D). In *cul-4(ok1891)* and *ddb-1(tm1769)* mutants, there was a delay in 5S loci pairing (53% and 49%, respectively) at the onset of meiosis (Zone 2) but reached higher levels of pairing, 80–90%, in pachytene (Zone 5; Fig 3C & 3D). This was also similar to what was previously shown for *csn-5(ok1064)* mutants ([34] and this study). Overall, the CRL4/CSN-Cop9 pathway mutations affect pairing, however the severity of pairing defects observed were milder compared to mutants involved in establishment of pairing (*e*.*g*. [21]).

In *C*. *elegans*, chromosomes are attached to the nuclear envelope by SUN/KASH domain proteins that associate with cytoplasmic Dynein, forming patches [49,52]. This leads to microtubule dependent chromosome movement at meiotic entry that is required for chromosome pairing and homologous synapsis. Mutants that abrogate this movement also lead to formation of PCs and delayed pairing. To test if the defects in pairing in CRL4 mutants stem from an inability to establish pairing interactions we assayed for formation of SUN-1 patches and chromosome movement. SUN-1 forms patches in early prophase nuclei in all genotypes tested, indicative of proficient clustering of pairing center proteins (S4 Fig,[52]). In agreement with lack of defects in SUN-1 patch formation, we observed no defects in chromosome movement (S4 Fig). This suggests that the impaired pairing outcome is due to an inability to stabilize pairing interactions and not due to defects in chromosome movement important for homolog pairing.

### Meiotic recombination is affected in CRL4 mutants

**Fig 4:**
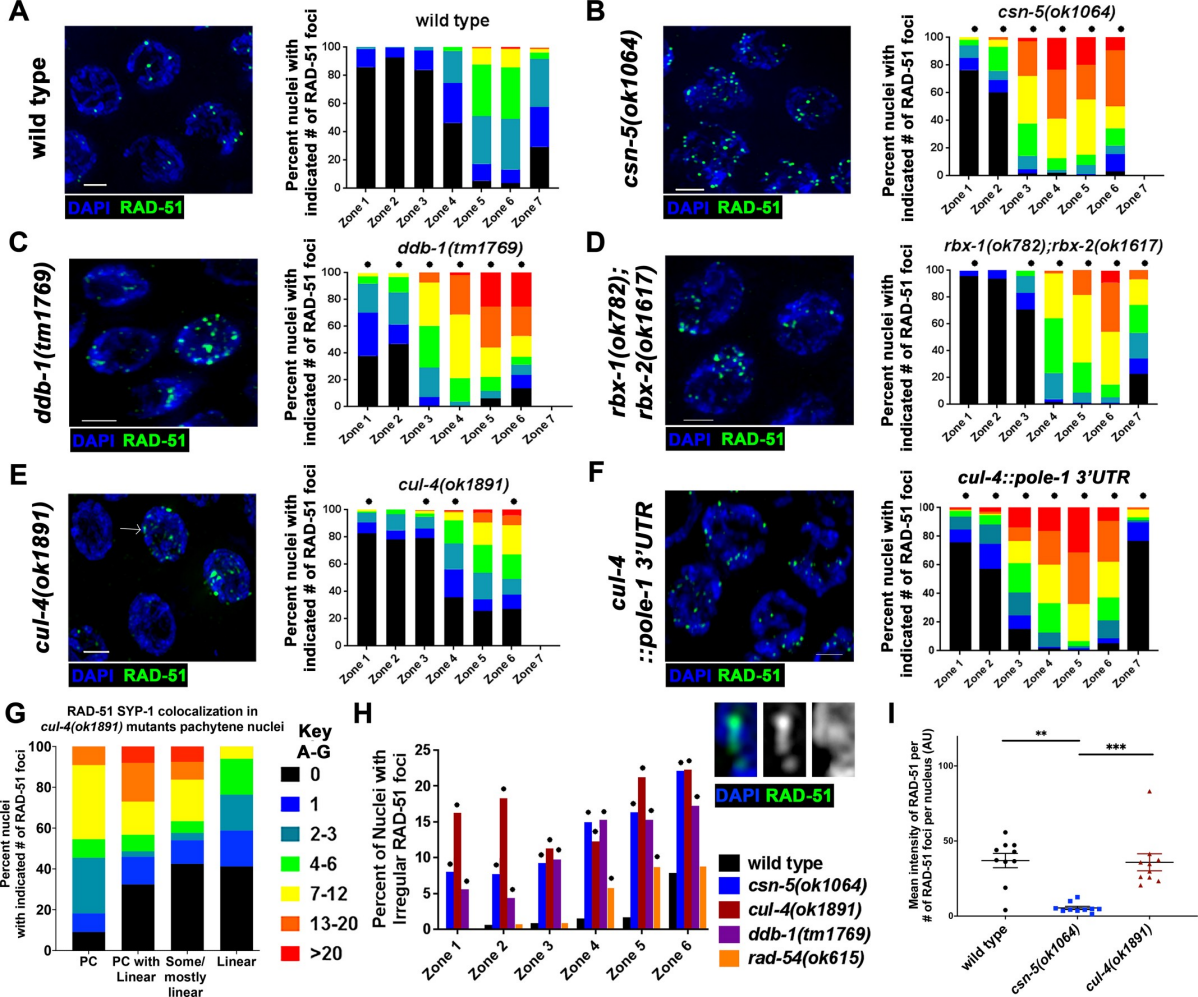
CRL4 complex mutants have increased numbers of meiotic recombination intermediates. A-F) Representative images of RAD-51 (green) immunofluorescent staining and DNA/DAPI (blue) from zone 5, left. Quantification of RAD-51 foci per nucleus throughout the germline in each genotype was designated into zones (discussed in Materials and Methods), right. Statistical comparisons were performed with wild type worms versus each individual CRL4 E3 ligase complex mutant (Mann-Whitney; p-values, * < 0.05). G) Number of RAD-51 foci in nuclei categorized based on SC assembly defects in *cul-4(ok1891)*. H) Irregular RAD-51 foci analyses, irregular foci are defined as long filamentous tracts or globular structures. Statistical comparisons were performed with wild type worms against individual mutants (Fisher’s exact test; p-values * <0.05). I) RAD-51 immunofluorescent intensity of foci: signal intensity of a nucleus was normalized to cytoplasmic RAD-51 immunofluorescence and divided by the number of RAD-51 foci quantified per nucleus. Each data point is one nucleus. For *cul-4* mutants, only nuclei with foci were analyzed (about half of the nuclei did not have any foci). Statistical comparisons were performed with wild type worms and individual mutants (Mann-Whitney; p-values, * <0.0005). Scale bars are 2μm.

Meiotic recombination in *C*. *elegans* requires the formation of functional SC. Previous studies have shown that PC formation involving proteins of the central region of the SC correlated with the accumulation of recombination intermediates in chromosomes, despite assembling intact lateral elements (*e*.*g*. [22]). These breaks are eventually repaired as sister chromatids become accessible for repair in late pachytene [53]. Single strand recombination intermediates can be visualized by antibody staining for RAD-51, which forms foci in meiotic nuclei starting in Zones 3–4 (Fig 4A). As has been shown previously, *csn-5(ok1064)* mutants have increased numbers of RAD-51 foci as nuclei progress through pachytene compared to wild type levels (Fig 4B, [34]). Similar to *csn-5(ok1064)* mutants, the CRL4 mutants *ddb-1(tm1769)* and *gad-1(ok573)* (Fig 4C; S5A Fig) showed an increase in RAD-51 foci in the germline. Interestingly, *rbx-1(ok782);rbx-2(ok1617)* mutants exhibited no observable defects in SC assembly (Fig 1G, S1G Fig), but had an increase in the amount of RAD-51 foci (Fig 4D). This effect is largely due to *rbx-1* since *rbx-1* (but not *rbx-2*) single mutants showed an increase in the amount of RAD-51 foci (S5C and S5D Fig). These results suggest that regulation of SC assembly may not be the sole function of the CRL4 complex and suggests that it is also involved in meiotic recombination. *cand-1(tm1683)* mutants did not show an increase in RAD-51 foci numbers compared to wild type (S5D Fig).

When RAD-51 foci numbers were analyzed, we found that the distribution of RAD-51 foci in *cul-4(ok1891)* mutants was unlike what was found in *csn-5(ok1064)*, *gad-1(ok573)*, or *ddb-1(tm1769)* mutants. We observed variation in the number of RAD-51 foci in Zones 4–6 where about 30% of the nuclei had no RAD-51 foci in *cul-4(ok1891)* mutants (Fig 4E). One explanation for this observation is that *cul-4(ok1891)* mutants have defects not only in DSB repair but also in DSB formation. A small fraction of *cul-4(ok1891)* mutant nuclei showed an increase in RAD-51 foci numbers, but not to the same extent found in *gad-1(ok573)*, *ddb-1(tm1769)*, or *csn-5(ok1064)* mutants. There is a correlation between the severity of the aggregation defects and the number of RAD-51 foci per nucleus in *cul-4(ok1891)* mutants; nuclei with PCs had more RAD-51 foci than nuclei with normal, linear SC (Fig 4G). Thus, it is possible that the defects in SC formation are responsible, in part, to the accumulation of RAD-51 foci in *cul-4(ok1891)* mutants, and this may be the same as in the other CRL4 mutants.

Along with the variation in the number of RAD-51 foci, there was an increase in irregular focus appearance in *cul-4(ok1891)* mutants (examples in Fig 4E & 4F). These irregular foci appeared as large lobed foci or string-like structures and were found in *cul-4(ok1891)*, *ddb-1(tm1769)*, and *csn-5(ok1064)* mutants (Fig 4H). These irregular foci were found throughout the germline, but their numbers increased as meiosis progressed. It is possible that irregular foci were a combined fluorescence of adjacent foci, which would be more common in mutants with high RAD-51 levels. If so, *rad-54* mutants, which are defective in RAD-51 unloading and exhibit elevated numbers of RAD-51 foci, should also have increased irregular foci. However, *rad-54* mutants have fewer irregular foci compared to *cul-4(ok1891)* mutants (Fig 4H), indicating that these foci are a product of improper meiotic recombination and its progression. Another possibility for the formation of irregular RAD-51 foci is an increase in nuclear RAD-51 protein. To examine this, fluorescence intensity of RAD-51 in nuclei was measured in *cul-4(ok1891)* and *csn-5(ok1064)* mutants in comparison to wild type nuclei. In *cul-4(ok1891)* mutants, intensity of RAD-51 fluorescence per nucleus was similar to that of wild type. In *csn-5(ok1064)* mutants, there was a significantly lower intensity of RAD-51 fluorescence per nucleus compared to wild type (Fig 4I). Thus, CUL-4 likely has a role in regulation of RAD-51, but not through RAD-51 degradation. Unlike the defects in SC assembly, which was found only in ~2/3 of the germlines analyzed, the aberrant accumulation of RAD-51 foci was found in all germlines examined (n = 39).

### CUL-4 promotes meiotic recombination and represses SPO-11 independent DSB formation

**Fig 5:**
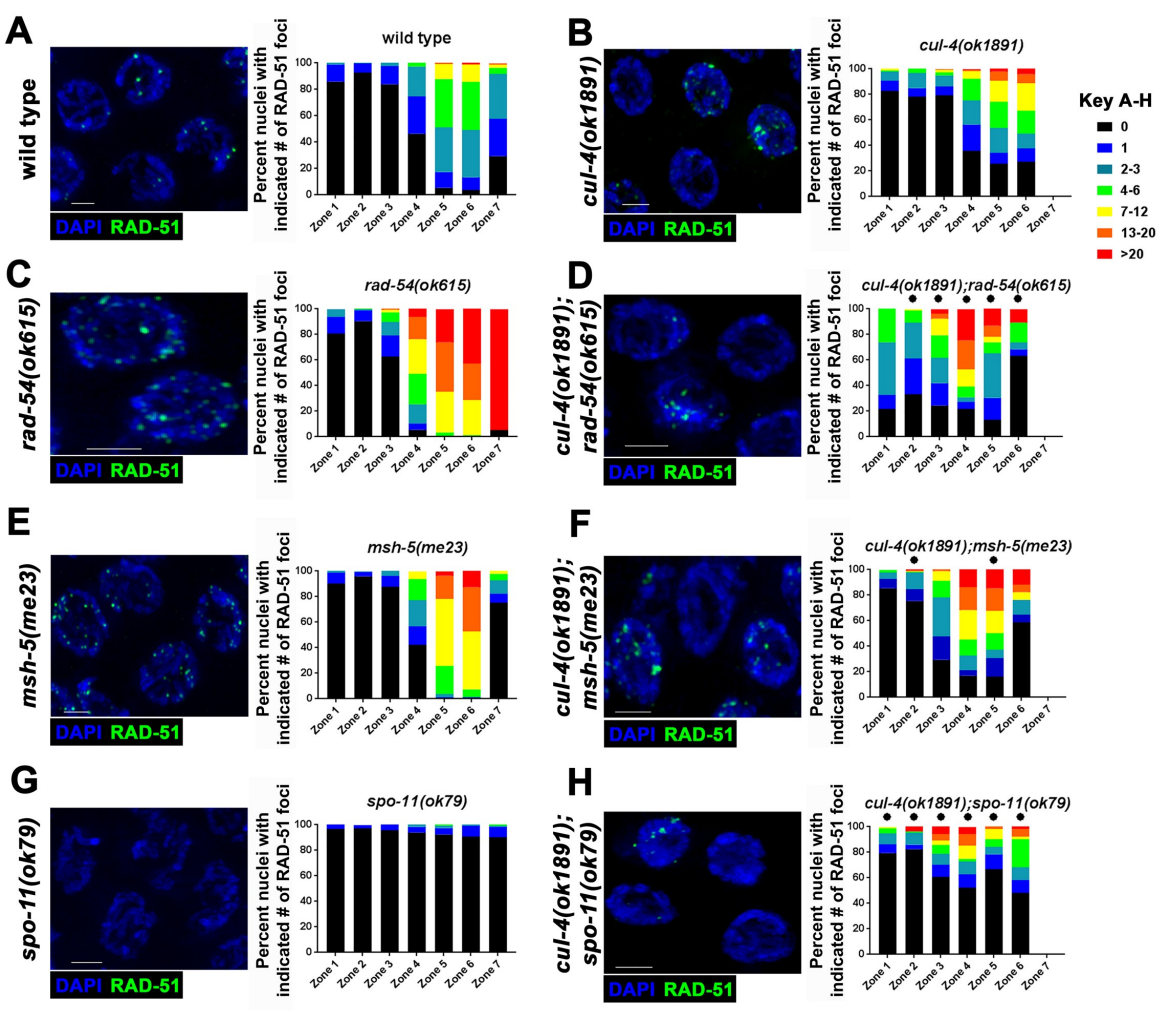
*cul-4(ok1891)* mutants have nuclei with decreased numbers of RAD-51 foci. A-H) Meiotic recombination intermediate (RAD-51) analyses in early meiotic recombination mutants in the *cul-4(ok1891)* mutant background. Representative images (left) are from Zone 5 (MP). Statistical comparisons were performed with double mutants versus single mutants (right; Fisher’s exact test; p-values, * <0.005). Scale bars are 2μm.

Because of the wide range of RAD-51 foci numbers in *cul-4(ok1891)* mutants, we tested whether nuclei with no RAD-51 foci may lack the ability to form SPO-11 generated DSBs. DSB formation and repair is a dynamic process, therefore the steady state number of RAD-51 foci in a given zone is an underestimation of the overall numbers of breaks generated. RAD-54 is required for DSB repair after the RAD-51 coated ssDNA invades the homologous sequence [54]. In the absence of RAD-54, RAD-51 filaments are not processed and the overall number of DSBs can be estimated from the number of RAD-51 foci. *rad-54(ok615)* mutants exhibit 24X increased levels of RAD-51 foci compared to wild type (Zone 7, Fig 5C). Interestingly, *cul-4(ok1891);rad-54(ok615)* double mutants had significantly lower numbers of RAD-51 foci in Zones 5 and 6 compared to *rad-54(ok615)* single mutants (Zone 5: 0.5X; Zone 6: 0.27X; Fig 5C & 5D). Approximately 13% of nuclei in Zone 5 of *cul-4(ok1891);rad-54(ok615)* double mutants had no observable RAD-51. On the other hand, *rad-54(ok615)* single mutants had no nuclei without RAD-51 foci supporting the idea that *cul-4(ok1891)* mutants have reduced numbers of DSBs (Fig 5B–5D).

To further support our hypothesis that some nuclei in *cul-4(ok1891)* mutants lack DSB formation, we examined *cul-4(ok1891);msh-5(me23)* double mutants. MSH-5 is important for crossover designation during recombination [53]. *msh-5(me23)* mutants have increased RAD-51 foci, but not to the extent of *rad-54(ok615)* mutants (Zone 6: ~47% *msh-5(me23)* ≥ 13 foci per nucleus, ~71% *rad-54(ok615)* ≥ 13 foci per nucleus; Fig 5C & 5E). The difference in phenotypes is due to the fact that DSBs can be repaired in *msh-5* mutants at late pachytene by homologous recombination producing noncrossovers ([53]; Fig 5E). In the double mutants, there was a population of nuclei that did not have RAD-51 foci and were similar to the *cul-4(ok1891);rad-54(ok615)* double mutants (Zone 5: *cul-4(ok1891);msh-5(me23)* = ~16% of nuclei; Fig 5D & 5F). These data suggest a role for CUL-4 in formation of SPO-11 induced DSBs.

Next, we tested if the DSBs formed in *cul-4(ok1891)* mutants leading to RAD-51 foci are dependent on SPO-11. In wild type cells, SPO-11 activity accounts for almost all germline DSBs and RAD-51 foci (Fig 4G). If DSBs in *cul-4(ok1891)* mutants were generated solely by SPO-11, then deletion of *spo-11* in *cul-4(ok1891)* mutants would eliminate the presence of RAD-51 foci. This was not the case, as throughout pachytene (Zones 3–6) approximately 40–50% of nuclei had RAD-51 foci (Fig 5H). The overall numbers of RAD-51 foci were reduced compared to *cul-4(ok1891)* mutants (Zone 6: *cul-4(ok1891)*- 5.2 foci/nucleus; *cul-4(ok1891);spo-11(ok79)*- 2.98 foci/nucleus; Fig 5B & 5H), but higher than observed in *spo-11(ok79)* mutants (Zone 6: 0.1 foci/nucleus; Fig 5G). To ensure that the immunofluorescent RAD-51 foci we observed were due to recognition of the RAD-51 protein, we analyzed RAD-51 focus formation in the *cul-4(ok1891)*;*rad-51(ok2218)* double mutants. As expected, no RAD-51 foci were observed in *cul-4(ok1891);rad-51(ok2218)* double mutants using the same methods (S5E & S5F Fig). We propose that *cul-4(ok1891)* mutants have a SPO-11 independent source of DSBs.

One possible explanation for the source of SPO-11-independent DNA damage is the movement of transposable elements. *C*. *elegans* transposable element movement is inhibited by the RNA interference pathway and by a set of mutator genes [12,55,56]. Approximately 12% of the *C*. *elegans* genome is made up of transposable elements (TEs), but only 6 TEs are found to be active, all of which are part of the *Tc1/Mariner* DNA TE class (reviewed in: [57]). Expression levels of two TEs, *Tc1* and *Tc3*, were examined by qRT-PCR. In wild type, *Tc1* had low levels to no levels of detectable expression. *Tc1* activity is known to be specific to somatic tissue [58], and therefore our results are likely basal expression readouts of somatic tissue transposition. In *cul-4(ok1891)* mutants, *Tc1* had no detectable expression, but *Tc3* had an average of 2.5-fold increased expression level compared to wild type (S5G Fig). Thus, in *cul-4(ok1891)* mutants, certain TE transposases are more highly expressed which could lead to increased mobility. An increase in *Tc3* transposition may be the SPO-11-independent source of DNA damage leading to the observed RAD-51 foci in the *cul-4(ok1891);spo-11(ok79)* double mutants. To test if loss of the CRL4 complex and not just perturbation of CUL-4 leads to increased expression of the *Tc3* transposase, we performed qRT-PCR of *Tc3* in *ddb-1(tm1769)* mutants. We observed a similar increase in *Tc3* expression in *ddb-1(tm1769)* compared to *cul-4(ok1891)* (**S5G Fig**), suggesting that perturbation of CRL4, and not just CUL-4, may lead to increased transposition.

### The CRL4 pathway is required for wild-type numbers of crossovers in meiosis

**Fig 6:**
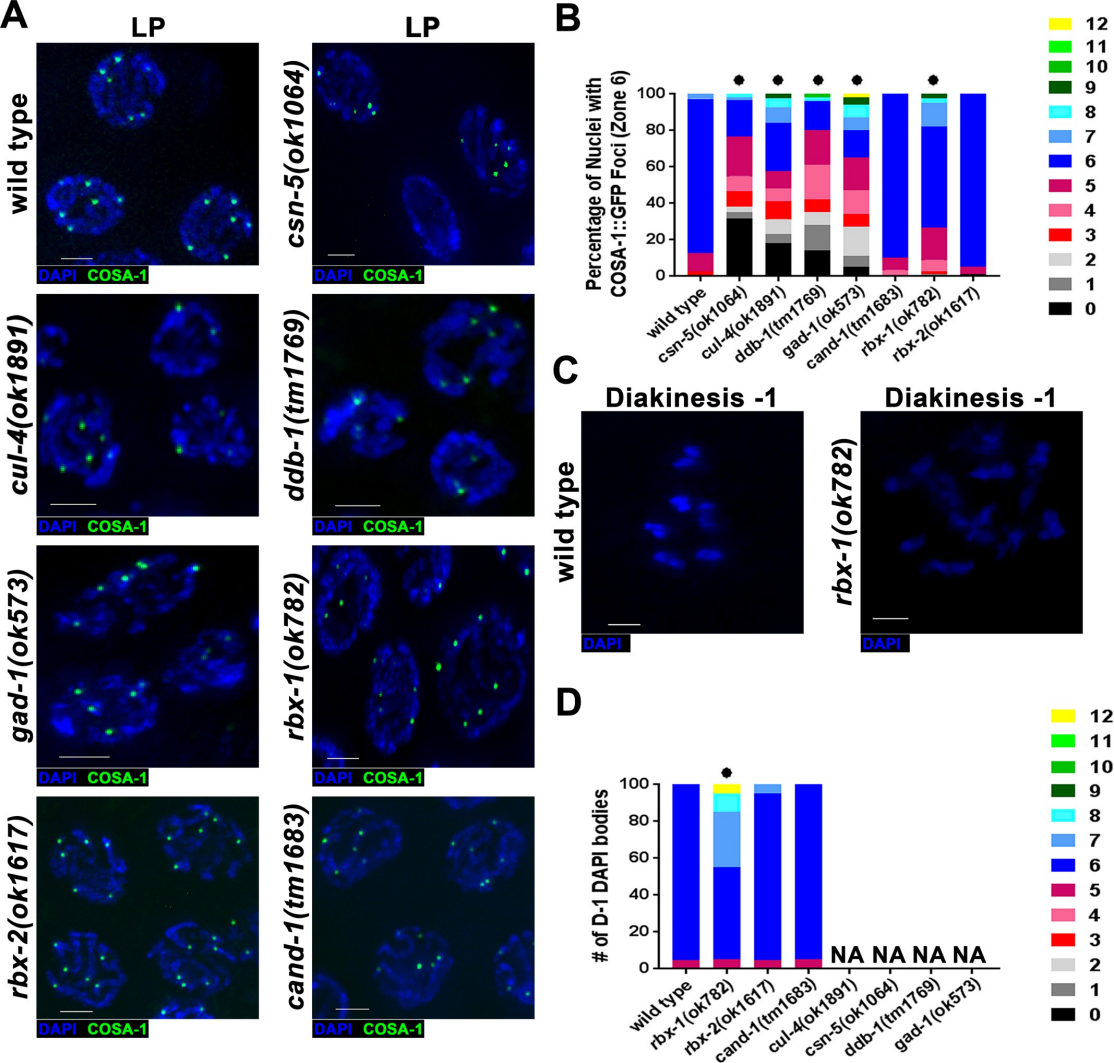
CUL-4 E3 ligase complex mutants likely affect the levels of crossovers. A) Representative late pachytene images of GFP::COSA-1 in CRL4 E3 ligase complex mutants. Blue (DAPI) and green (COSA-1::GFP) B) Quantification of GFP::COSA-1 per nucleus of each different genotype, images analyzed were from Zone 6/7 (dependent upon the length of germline). Statistical analyses were performed in comparison to wild type worms (Mann-Whitney; p-values, * < 0.0005). C) Representative images of diakinesis-1 nuclei in wild type (6 DAPI bodies) and *rbx-1(ok782)* mutants (12 DAPI bodies), blue (DAPI). D) Quantification of the number of DAPI bodies at D-1 in each different genotype. NA = Not Applicable due to these mutants not progressing past pachytene. Statistical analyses were performed in comparison to wild type worms (Mann-Whitney; p-values, * < 0.001). Scale bars are 2μm.

We have previously shown that the CSN/Cop9 signalosome is required for crossover formation [34]. Crossovers are divided into two subgroups: interfering (non-randomly spaced) and non-interfering crossovers (randomly spaced). In the *C*. *elegans* wild type germline all crossovers are interfering and are marked by COSA-1, forming one focus per chromosome [59]. To determine if CRL4 components are also required for crossovers, CRL4 mutants were crossed into a *gfp*::*cosa-1* transgenic background. Mutants of the CRL4 complex (that also exhibit PC formation), on average, had lower levels of GFP::COSA-1 foci as compared to wild type (~61–77% to that of wild type; Fig 6A & 6B). There was also a small population of nuclei with increased levels of GFP::COSA-1 in CRL4 mutants (up to 20%; Fig 6B). This could be due to incomplete stabilization of crossover sites with COSA-1 localizing to multiple sites, indicative of meiotic recombination delay. In meiotic prophase I, progression of nuclei past pachytene are defined as part of the diplotene/diakinesis stage. CRL4 mutants, and *csn-5(ok1064)* mutants, do not progress to this stage; instead meiotic progression halts at late pachytene (Zone 6). This is true for all CRL4 mutants except for *rbx-1* mutants. *rbx-1* mutants have nuclei which progress into the final stage of diakinesis called D-1. Nuclei at this stage have 6 DAPI bodies representing the six bivalents (Fig 6C & 6D). *rbx-1* mutants had an average of 6.8 DAPI bodies indicating that chiasma in some nuclei had not occurred or had been lost (Fig 6C & 6D), suggesting a minor role for RBX-1 in crossover formation.

### PC formation in *cul-4(ok1891)* mutants is dependent on SPO-11 generated DSBs

**Fig 7:**
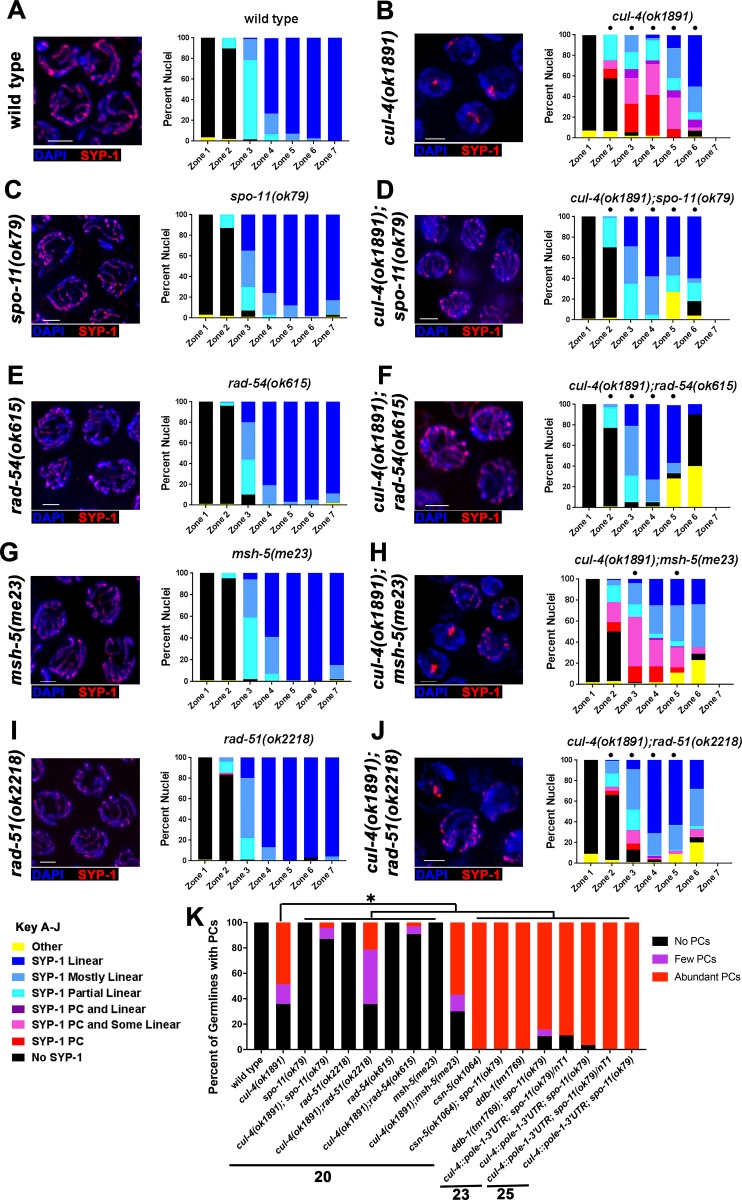
PC formation in *cul-4(ok1891)* mutants is dependent upon the presence of early meiotic recombination proteins. A-J) SYP-1 analysis of SC assembly in meiotic recombination mutants in a *cul-4(ok1891)* mutant background (“No SYP-1” was defined as having no SYP-1 immunofluorescence present along chromosomes (DAPI). “SYP-1 PC” were nuclei with only PC formation present, no elongation of SYP-1 along DAPI. “SYP-1 PC and Some Linear” was the presence of PC(s) in the nucleus but also partial elongation of SYP-1, <50% of DAPI. “SYP-1 PC and Linear” was similar to “SYP-1 PC and Some Linear” with the exception that SYP-1 is elongated along >50% of DAPI. “SYP-1 Partial Linear” nuclei had elongated SYP-1 along up to 50% of DAPI. “SYP-1 Mostly Linear” nuclei had SYP-1 along up to 50% of DAPI but less than 100%. “SYP-1 Linear” nuclei had fully elongated SYP-1 along all DAPI. “Other” was defined as nuclei that had abnormal DAPI appearance). Left: representative images of Zone 4 (EP) in each genotype; right: quantification of SYP-1 morphology throughout the germline of each genotype. Statistical comparisons were performed with double mutants versus single mutants (Fisher’s exact test; p-values, * < 0.05). Scale bars are 2μm. K) Analysis of PC presence in all germlines analyzed for each genotype. The number of PCs per germline were grouped into three main categories: No PCs = germlines with no SC PC formation, Few PCs = germlines no more than 5 PCs or less than ½ of a zone with PCs, and Abundant PCs = germlines with more than 5 PCs or greater than ½ of a zone with PCs. Statistical comparisons were performed against *cul-4(ok1891)* mutants. Statistics: Fisher’s exact test; p-values, * < 0.001, except *cul-4; rad-51* that was significant only using 2x3 contingency table the Freeman-Halton extension of Fisher's Exact test (to calculate differences between 3 categories: No PCs x Few PCs x Abundant PCs).

It has been proposed that SC assembly is unaffected by the presence or progression of recombination in *C*. *elegans*. For example, SC assembles in *spo-11* mutants as proficiently as in wild type ([6]; Fig 7C). Surprisingly, in *cul-4(ok1891);spo-11(ok79)* double mutants, PC formation was almost completely eliminated (Fig 7D). Formation of SC between chromosomes does not necessarily mean that homologous chromosomes are synapsed, as SC can form aberrantly between non-homologous chromosomes in some mutants [17]). To determine if *spo-11(ok79)* suppressed the *cul-4(ok1891)* mutants truly, restoring homologous synapsis, we performed SYP-1 staining combined with FISH (5S locus). We observed that 94.5% of pachytene nuclei in *cul-4(ok1891);spo-11(ok79)* double mutants (n = 290) contained 5S paired foci flanking SYP-1 linear stretches, indicative of homologous synapsis. This frequency was slightly lower than what was found in wild type (98.7% n = 371, p = 0.003 Fisher’s Exact Test). This indicates that the majority of chromosomes in pachytene nuclei of *cul-4(ok1891);spo-11(ok79)* double mutants were homologously-synapsed. It is possible that DSB processing/repair defects and not only DSB formation are required for PC formation in the *cul-4(ok1891)* mutants. To test this, we examined *rad-54(ok615)* mutants in the *cul-4(ok1891)* mutant background. Similar to *cul-4(ok1891);spo-11(ok79)* double mutants, there was a significant decrease in the amount of PC formation in *cul-4(ok1891);rad-54(ok615)* mutants (Fig 7F).

To determine the point at which meiotic recombination no longer affects PC formation, we assessed *cul-4(ok1891);msh-5(me23)* double mutants expecting no reduction in PC formation if only early recombination components had an effect. In these double mutants, we found that PC formation was similar to *cul-4(ok1891)* single mutants (Fig 7H). Therefore, PC formation in *cul-4(ok1891)* mutants is dependent upon DSB formation and early meiotic recombination progression but not later recombination events. *cul-4(ok1891);rad-51(ok2218)* mutants were also analyzed to identify if the recombinase RAD-51 is required for PC formation in *cul-4(ok1891)* mutants. Interestingly, PC formation occurs in *cul-4(ok1891);rad-51(ok2218)* double mutants, but to a lesser degree than in *cul-4(ok1891)* single mutants (~30% and 95% in Zones 3 and 4, respectively and 23% of germlines with abundant PCs compared to 49% in *cul-4* single mutants; Fig 7B & 7J). Thus, the initiation of homologous recombination through SPO-11, and in part, the formation of recombination intermediates is required for PC formation in *cul-4(ok1891)* mutants (Fig 7K depicts the percentage of germlines with abundant, few, or no PCs). We did not observe suppression of PC formation by *spo-11* in *cul-4*::*pole-1-3’UTR* or *ddb-1(tm1769)* (Fig 7K) indicating that the effect is specific to the *cul-4(ok1891)* mutants. Regardless, our data demonstrates that PC formation can be dependent on repair of SPO-11 generated DSBs, under some circumstances, and PC formation is not necessarily uncoupled from recombination as previously thought.

## Discussion

In this study, we examined the mutant phenotypes of the CRL4 E3 Ligase complex in the context of meiotic prophase I in the *C*. *elegans* germline. All mutants tested in genes encoding for the CRL4 E3 ligase complex exhibited PC formation except for *rbx* mutants (see model in S6A Fig). Interestingly, PC formation in *cul-4* C’ terminal truncation mutants was dependent on early recombination. Mutants of CRL4 also had increased levels of RAD-51 foci, indicating delays in meiotic recombination. *cul-4* mutants had a mixed population of nuclei that either had no RAD-51 foci or increased levels of RAD-51 foci. Due to these recombination defects, crossovers in CRL4 mutants were at lower levels. CRL4 also may play a role in germline protection from TE movement as mutants have increased expression of the *Tc3* TE. Our data indicate that CRL4 plays multiple roles in meiotic prophase I for SC assembly, meiotic recombination, and the preservation of germline integrity.

### The CRL4 E3 Ligase complex is required for preventing SC protein aggregation into PC structures

PCs can be categorized based on their reaction to 1,6-Hexanediol, a chemical that dissolves weak hydrophobic interactions. In the context of the PC, the ability to be dissolved by 1,6-Hexanediol, was interpreted as an indication that molecules can move rapidly in and out of the PC structure, similarly to what is found in a wild type PC. The inability to dissolve the PC by 1,6-Hexanediol indicated that the structure is rigid. The nature of this rigid structure is open to debate. In *C*. *elegans* PCs can be formed in wild type germlines, at elevated temperatures [60]. These heat induced PCs were shown to be resistant to 1,6-Hexanediol treatment, and therefore likely rigid [48]. In contrast, PCs formed by precocious self-assembly of the SC proteins [61–64], defects in lateral element formation [18], and perturbation of chromosome movement [49,50] likely still retain the dynamic characteristic of wild type SC, as PCs in some of these mentioned perturbations were shown to be sensitive to 1,6-Hexanediol. CRL4 is likely involved in prevention of formation of the rigid PC structure as 1,6-Hexanediol was unable to disperse the PCs formed in CRL4 mutants (*cul-4*, *gad-1*, *ddb-1*, or *csn-5* mutants). PC formation in CRL4 mutants also exhibited temperature-dependence as the number of SC PCs increased at elevated temperatures [*cul-4*, *gad-1*, *ddb-1*, as well as the partial loss-of-function mutant of *cul-4* (See Fig 2 and model in S6B Fig)].

Two possible explanations exist as to why PC structures are rigid/1,6-Hexanediol-resistant in CRL4 mutants. One possibility is that SC proteins are changing their structure in a way that causes decreased dynamics. It cannot yet be determined if this change in structure is minimal (conformational change) or severe (misfolded protein). This model suggests that CRL4 promotes directly, or indirectly, protein structure of one or more of the SYP proteins. These mis-shaped proteins can be degraded or refolded as nuclei progress through prophase, as most nuclei do not contain PCs in late pachytene. The movement of nuclei in the germline is approximately 1 row per hour which leaves significant amount of time for the process of protein degradation/refolding (estimated ~10 hours from mid to late pachytene). Alternatively, in the absence of CRL4, altered protein modifications can lead to a rigid SC structure by adding/removing binding partners, or generating a conformation different from wild type. Due to the temperature sensitivity of the phenotype and the lack of direct evidence for modifications of SYP proteins in *cul-4* mutants we prefer the first model, but both models are plausible. Our ability to detect a small population of modified SYP by this assay may be difficult, but according to our current data, the role of CRL4 appears to be indirect.

A number of post-translational modifications have been shown to target and affect SC proteins. One of the most iconic is sumoylation of Ecm11 in yeast, important for both SC assembly and recombination [65–67]. In mouse and yeast, it was shown that both SUMOylation and ubiquitination play an important role in SC assembly and recombination progression, both through proteasome recruitment to chromosome axes [33,68]. SUMOylation plays no role in SC assembly in *C*. *elegans* [69,70]; instead, SYP-1 acetylation [71] and phosphorylation [72], are important for assembly and disassembly, respectively. Phosphorylation of SYP-4 was found to be important for changing the structure of the SC from a dynamic nature, where SC proteins are mobile, to one that is less dynamic [23]. We provide evidence that another post-translational modification, ubiquitination, affects SC assembly in *C*. *elegans* ([34]and this manuscript). This is in agreement with studies demonstrating that proteasome inhibition leads to PC formation in mouse and *C*. *elegans* [33,68]. Like our study, these studies did not demonstrate a direct effect of ubiquitination on SC proteins.

We have shown that mutants of *csn-5*, *cul-4*, *ddb-1*, and *gad-1* all lead to similar phenotypes: defects in SC assembly accompanied by PC formation. We have shown that the PCs formed in CRL4 mutants are specific to central region proteins and do not affect axial element proteins of the SC. However, we found no evidence for ubiquitination of SYP proteins, as no reproducible change in molecular mass was observed in SYP proteins in mutants of either *cul-4(ok1891)* or *csn-5(ok1064)*. This suggests that the effect of ubiquitination on SC formation is indirect. It is possible that proteins involved with regulating the rigidity of the SC may be targeted for ubiquitination.

In previous work, we have shown that mutants of the CSN/Cop9 complex have defects in SC assembly [34]. Based on the similarity of phenotypes observed between *cul-4* mutants and CSN/Cop9 mutants and studies in other systems (reviewed in: [73]), we propose that CSN/Cop9 deneddylates CRL4 (Fig 1A). After deneddylation, CRL4 then targets another protein(s) for ubiquitination that indirectly maintains the liquid property of the SC, which is required for SC assembly. The current model of CRLs states that their neddylated form is the active form, and the lack of deneddylation prevents the recycling of the E3 ligase complex and/or its stability [74]. Based on this model, an increase in neddylation (*e*.*g*., *csn-5* mutants) or loss of CRL4 activity (*e*.*g*., *cul-4*, *gad-1*, *ddb-1* mutants), both lead to the same outcome. The data we present here are consistent with this model.

Another interesting finding is that the *rbx* genes have no role in SC assembly, which does not fit the canonical CRL4 complex model (Fig 1A). It is possible that RBX-1 acts solely as a member of CRL4 that is involved in DNA repair, as we have no evidence supporting its function in SC assembly, and instead see a difference in recombination intermediate abundance (RAD-51). Both *rbx* mutants are expected to be null based on the fact that *rbx-1(ok782)* deletes the whole gene and *rbx-2(ok1617)* deletes half of the coding frame. RBX-1 and RBX-2 are the only ROC1 homologs in *C*. *elegans*. It is possible that other functional homologs exist that are unidentifiable by sequence homology or that CRL4 in *C*. *elegans* can interact with other RING domain proteins. The RING domain proteins acting in CRLs are small and part of the H2 subfamily of RING finger proteins [73]. The *C*. *elegans* genome encodes for 101 other RING proteins, any of which may act redundantly with *rbx-1/2* (Moore & Boyd, 2004). Consistent with these possibilities, *rbx-1(ok782)*;*rbx-2(ok1617)* mutants progress through larval stages, while *cul-4* null mutants cannot. Alternatively, CUL-4 may function in regulation of PC formation independently from its activity as an E3 ligase. This is supported by the fact that *cul-4(ok1891)* mutants which have a C’ truncation that removes the RBX binding site, can support SC formation in ~35% of germlines at 20°C. According to this model, the phenotypes associated with *cul-4(ok1891)* mutants are attributed to the reduction in CUL-4 levels and not to the removal of the RBX domain. As far as we know, there is no evidence for CRL4 acting outside of its role as an E3 ligase, which may favor our first model (alternative RING protein to RBX-1/2).

The role of CAND-1 is to sequester the Cullin complexes once they are deneddylated to prevent over-activation via rapid neddylation. In terms of PC formation, *cand-1* mutants have a relatively mild phenotype in comparison to other mutants of the CRL4 E3 ligase complex, which is consistent with a previous study where defects of *cand-1* mutants mildly mimicked Cullin mutant phenotypes [46]. Along with the fact that *cand-1* homozygous mutants are fertile; these data suggest that *cand-1* activity is only partially impactful where Cullin activity occurs. We conclude that deactivation of Cullin ligases by deneddylation associated with the binding of the CSN/Cop9 complex is more important for controlling CRL4 activity in the germline than sequestering of the deneddylated ligases with CAND-1, indicating that quick recycling of CRL4 is key for SC assembly.

### CRL4 is required for proper progression of recombination

In our previous work, we had shown that mutants of the CSN/Cop9 complex have defects in recombination that were attributed to defects in SC assembly [34]. Since CSN/Cop9 acts through CRL4, the same logical argument can be applied here. Lack of assembly of the central region of the SC led to defects in recombination; DSBs are formed but cannot be repaired though homologous recombination with the homologous chromosome [For example: [22,50,75]]. Thus, PC formation in CRL4 mutants may explain why RAD-51 foci accumulate in these mutants and crossover levels are reduced. Unlike what is found for SYP null mutants [20–22,75], CRL4 mutants still show ~50–70% homologous synapsis which explains why crossover levels are reduced but not eliminated in these mutants. This may be the most parsimonious explanation for the results. However, our data suggests that this may not be a complete explanation. *rbx-1;rbx-2* double mutants that have no PC formation still exhibit a small but significant elevation in RAD-51 foci, compared to wild type. This may indicate that the CRL4 complex in which RBX-1/2 are not participating regulates SC assembly, while the CRL4 complex in which RBX-1/2 are present has additional function(s) in DSB repair. Since *rbx-1* mutants show only a mild effect on COSA-1 foci numbers, this indicates that the direct effects of the CRL4 complex in DSB repair are minor compared to the indirect effects of SC assembly.

CUL4 has an established function in somatic DSB repair and DNA damage signaling. After UV damage, CUL4 plays a role in recognition and nucleotide excision repair of damage as well as halting of cell cycle progression after this damage [76,77]. CUL4 signals for DNA damage recognition through ubiquitination of histones, H2B, H3, and H4, which has been shown to facilitate Exo1-mediated resection [78,79]. CUL4 also facilitates DNA damage recognition by stabilization of p53 through PCNA interactions [37], and CRL4 inactivation leads to the induction of p53 [80]. Upstream of p53 in DNA damage recognition and signaling is the Chk1 kinase. Chk1 has also been shown to be regulated by CUL4 in both normally cycling cells and cells under replication stress [81]. If CRL4 directly affects repair of DSBs, the appearance of irregular RAD-51 foci in *cul-4* and *ddb-1* mutants may be a cytological manifestation of a direct effect on recombination.

### Truncation of the C’ terminal domain of CUL-4

While *ddb-1(tm1769)* and *gad-1(ok573)* are likely null alleles, *cul-4(ok1891)* confers a partial loss-of-function. *cul-4(ok1891)* mutants produce viable adults in low frequency, while two other mutants of *cul-4*, *gk434* and *gk511*, arrest in the larval stages. Why *ddb-1* and *gad-1* null mutants still progress through development unlike the null *cul-4* allele may be explained by redundancy: CRLs are known to work with many alternative adaptor/substrate recognition pair(s) [36]. Thus, DDB-1 and GAD-1 may have a more important function in the germline than in larval development.

The *ok1891* allele is a deletion located near the C-terminus of the protein, leading to a frameshift mutation; while *cul-4(gk434)* and *cul-4(gk511)* are deletions predicted to cause earlier truncations in the coding sequence. The *ok1891* deletion removes the neddylation target site and also the binding site for the E2 enzyme. Therefore, the *cul-4(ok1891)* mutation likely affects CUL-4’s ability to form a fully functional CRL4 complex. However, the fact that some worms of *cul-4(ok1891)* mutants [but not *cul-4(gk434)* and *cul-4(gk511)* mutants] do not trigger larval arrest, indicates that this malformed CRL4 complex maintains partial activity circumventing the essential function of CUL-4 during larval development. It is possible that in the absence of the lysine modified by neddylation in wild type CUL-4, another lysine serves as an alternative site for modification in *cul-4(ok1891)* mutants, rendering it partially active. It is perplexing how CUL-4 protein that lacks the C’ domain (RBX/ROC-E2 binding) was able to bypass the need for a functional CRL4 in some worms. It is possible that the E2 enzyme can modify some CRL4 substrates without directly binding to CUL-4; the C’ truncated form of CUL-4 that is still able to bind the substrate may provide some assistance to the E2 creating a partial loss of function phenotype.

*cul-4*::*pole-1 3’UTR* have similar increased levels of RAD-51 as to these observed in the *csn-5*, *ddb-1*, and *gad-1* mutants. However, in *cul-4(ok1891)* mutants, meiotic prophase I nuclei have SPO-11 independent DSBs as well as nuclei that lack SPO-11 DSBs. Moreover, in *cul-4*::*pole-1 3’UTR* mutants, PC formation is independent of SPO-11, whereas in *cul-4(ok1891)* mutants PC formation is dependent on SPO-11. These findings indicate that germline nuclei of these two different *cul-4* mutants did not produce the same defects, aside from PC formation, and this can be explained by a gain-of-function in the *cul-4(ok1891)* allele. The gain-of-function phenotypes in *cul-4(ok1891)* mutants may be due to the formation of an aberrant CRL4 complex (that binds the substrate through DDB-1/GAD-1, but not the E2) that interrupts SPO-11 induced DSB formation. Alternatively, the unique phenotypes for *cul-4(ok1891)* mutants compared to *cul-4*::*pole-1 3’UTR* mutants may be due to a manifestation of a more severe reduction in CUL-4 levels. In this model, CUL-4 forms a complex with other CRL4 complex proteins, beside GAD-1/DDB-1, and performs germline functions outside SC assembly. In this case, CUL-4 activity in SC assembly (GAD-1/DDB-1 dependent) is perturbed due to the reduction of CUL-4 availability, however other CUL-4 activities such as DSB -dependent PC formation and transposition, are only abrogated when CUL-4 levels drop even further. This may explain why *cul-4*::*pole-1 3’UTR* mutants (~70% of wild type protein levels) are similar to *ddb-1* and *gad-1* mutants, while *cul-4(ok1891)* mutants (~15% of wild type protein levels) exhibit distinct phenotypes (S6A Fig). In this model, different CRL4 complexes have different required CUL-4 proteins levels for proper function. The caveat with this model *vs*. the gain-of-function activity model is that it assumes that the C’ truncated CUL-4 is at least partially functional, retaining E3 ligase activity.

Another unique feature of *cul-4(ok1891)* mutants particularly in comparison to *csn-5*, *ddb-1*, and *gad-1* mutants is the incomplete penetrance of the PC formation phenotype. PCs were present in 2 out of 3 germlines analyzed and two germlines of the same worm may exhibit different phenotypes. The high variation in levels of nuclear localization of CUL-4 in *cul-4(ok1891)* mutants may explain the incomplete penetrance observed at 20°C. Similarly, *cul-4*::*pole-1 3’UTR* mutants also shows partial penetrance, but is observed at higher temperatures (22–24°C). In *cul-4*::*pole-1 3’UTR* mutants, CUL-4 levels are reduced but not to the same extent as *cul-4(ok1891)* mutants. This may explain why PC formation only appears at higher temperatures in *cul-4*::*pole-1 3’UTR* mutants.

### Interplay between SC assembly and recombination in *C*. *elegans*

In organisms such as mouse or yeast, SC assembly depends on the initiation of meiotic recombination by SPO-11 [7,8]. In the absence of SPO-11 in *C*. *elegans*, the SC assembles along homologous chromosomes similar to wild type [6]. Thus, recombination is not typically seen as an interfering process in SC assembly; it either has a positive or neutral effect. In *cul-4(ok1891)* mutants, PC formation is reduced when early recombination is inhibited (*ex*. loss of SPO-11 or RAD-54), but no such effect was observed when CRL4 levels are reduced by 3’UTR replacement or by removing *ddb-1*, despite high penetrance of the PC-formation phenotype. This suggests that in the presence of functional CRL4, crosstalk between recombination and PC assembly is prevented in *C*. *elegans*, but aberrant CRL4 formation or its very low levels (in *cul-4(ok1891)* mutants) leads to a link, or crosstalk, between PC formation and recombination.

The only other known example of crosstalk between recombination and SC assembly in *C*. *elegans* is in the *cra-1* mutant background where loss of SPO-11 caused increased assembly defects [25]. CRA-1 is a NatB domain-containing protein shown to have roles in synapsis and crossover formation on autosomes as well as global histone acetylation [25,82]. In *cra-1* mutants, SC assembly defects become more severe in the absence of early meiotic recombination intermediates [25]. Thus, CRL4 prevents recombination interfering with SC assembly and CRA-1 inhibits some mechanism that promotes SC assembly through recombination.

Another important connection between SC and recombination was revealed in studies of SC dynamics. Despite the fact that the SC may appear as a rigid structure, SC proteins were shown to be exchanged in a dynamic manner throughout early to mid-prophase [23,24]. This exchange was attenuated in late prophase and is dependent on the formation of crossovers [24]. These findings imply that the recombination process may negatively regulate SC assembly by limiting the influx of new SYP proteins to the SC. Unlike CRL4 mutants which mainly affect early to mid-prophase, the effect on SC dynamics is restricted to late pachytene. However, if PCs can be considered a less dynamic SC structure than assembled SC, then our studies and that of [23] both show similar effect that are executed at different time points in meiotic prophase I. Both studies show that recombination can promote a less dynamic SC structure, but depending on the point in prophase can lead to a different effect: PC formation in early prophase, while promoting SC disassembly in late prophase. This may also relate to the desynapsis pathway that opposes SC stability after the SC is formed. In this pathway, recombination intermediates destabilize the SC for chromosomes in which not all crossovers have been formed, leading to SC disassembly but without PC formation [83]. However, this pathway is not only phenotypically distinct but also known to act later in prophase, whereas CRL4 acts much earlier in prophase during SC assembly.

In *cul-4* C-terminal truncation mutants, PC formation is reduced when recombination is prevented (loss of SPO-11) or inhibited at its early steps (loss of RAD-54 and to a lesser degree RAD-51). Loss of the pro-crossover factor MSH-5 had no effect on PC formation. This indicates that PC formation is inhibited when recombination intermediates (RAD-51 covered ssDNA) do not form or when recombination intermediates are locked in position (form joint molecules but are unable to disassemble). However, the effect of removing RAD-51 on PC formation in *cul-4(ok1891)* mutants is milder than that of removing RAD-54. This suggests that the mechanism involved responds to perturbation of recombination at different levels, when perturbation of early (*spo-11*) or late (*rad-54*) events in recombination is more important. In this model, the RAD-54 protein itself may directly participate in the PC formation. Alternatively, RAD-51 may be required for amplification of the signal caused by impaired recombination, proposing that in *rad-51* mutants the signal existed but cannot be transmitted. As discussed above, some aspects of DSB repair defects in CRL mutants may be independent of PC formation, thus it is plausible that CRL4 has more than one target for ubiquitination; one target that promotes formation of functional recombination intermediates and another target involved in SC assembly.

### CUL-4’s multiple roles in meiotic prophase I

In previous studies, CUL4 has been shown to play multiple roles in meiotic prophase I. In mouse and the Chinese mitten crab, loss of CUL4 led to increased levels of apoptosis, decreased and malformed spermatozoa, and a delay of meiotic recombination with accumulation of late recombination intermediates [29,41,84]. This study provides further evidence that CUL-4 ubiquitination activity has an important function in meiosis. Despite the clear importance of CRL4 in meiosis, the question remains as to the identity of CRL4 targets in meiotic prophase I.

CUL-4 plays an important role in cell cycle progression and meiotic entrance. CUL-4 prevents re-replication in S-phase via ubiquitin-signaled degradation of CDT-1, a replication licensing factor. This function is well conserved and is present in humans, mice, frogs, worms, flies, and yeast (reviewed in: [43]). In *C*. *elegans*, CRL4 was found be important for export of CDC-6, another replication licensing factor that complexes with CDT-1 [39]. We have shown that CSN/Cop9 mutants have proliferation defects that account for the smaller germlines observed in these mutants [34], and CRL4’s function in replication licensing may explain this phenotype.

We have shown here that CRL4 acts in SC assembly and has both direct and indirect roles in recombination. Through the analysis of *cul-4(ok1891)* mutants, we have shown that dysfunctional CRL4 can interfere with both DSB formation and downregulation of SPO-11 independent DSBs. Loss of CUL-4 could affect localization or function in a number of different proteins important for DSB formation. Another possibility is that DSB formation defects stem from CUL-4’s effect on chromosomal structure, since CUL-4 is known to ubiquitinate histones in humans and mice (discussed in: [85]). Our analysis also demonstrated that dysfunctional CRL4 can interfere with the repression of the *Tc3* transposase. While we have not directly measured mobility due to the sterility of CRL4 mutants, we suspect that it is increased; if so, transposon movement could contribute to the SPO-11 independent DSBs observed in the *cul-4* mutant. [13] have shown that the PIWI pathway downregulates *Tc3* expression and has no role in the regulation of *Tc1* transposase expression [13]. Therefore, one explanation for CRL4 mutants’ effects on *Tc3* transposase expression would be the mis-regulation of PIWI.

To conclude, we propose that CRL4 plays a central role in meiotic prophase events; it is required for maintaining the functional and dynamic SC, and its proper activity promotes formation of recombination intermediates that do not interfere with SC assembly. Dysfunctional CRL4 can interfere with DSB formation and activate transposition. Future studies should aim to identify the targets of CRL4 in meiosis.

## Materials and methods

### Strains

*C*. *elegans* strains were cultured at standard conditions at 20°C (Brenner, 1974). The wild type background strain used was N2 Bristol. The following mutations and chromosome rearrangements were used: LGI: *akir-1(gk528)*, *htp-3(tm3655)*, *rad-54&snx-3(ok615)*, *rbx-2(ok1617)*, *rrf-1(pk1417)*, *hT2[bli-4(e937) let-*?*(q782) qIs48] (I;III)*; LGII: *cul-4(ok1891)*, *mIn1 [mIs14 dpy-10(e128)] II*; LGIV: *csn-5(ok1064)*, *ddb-1(tm1769)*, *msh-5(me23)*, *rad-51(ok2218)*, *spo-11(ok79)*, *spo-11(iow110)*, *nT1[qIs51] (IV;V);* LGV: *cand-1(tm1683)*, *gad-1(ok573)*, *rbx-1(ok782)*, *nT1[qIs51] (IV;V)*.The following transgenic lines were used: *syp-4(iow28[V5*::*syp-4]) I*, *cul-4(iow64[cul-4*::*pole-1-3’UTR]) II*, *meIs8[pie-1p*::*GFP*:: *cosa-1+unc-119(+)] II*, *meIs9[unc-119(+) pie-1promoter*::*gfp*::*SYP-3];unc-119(ed3) III*, *syp-3(iow69[3xFLAG*::*syp-3]) III*, *ddb-1(iow60[OLLAS*::*ddb-1]) IV*, *gad-1(iow61[3xFLAG*::*gad-1]) V*, *syp-1(iow68[syp-1*::*3xFLAG]) V*, *syp-2(iow27[syp-2*::*V5]) V*.

### CRISPR/Cas9 generation of *syp-1*::*FLAG*, *syp-2*::*V5*, *FLAG*::*syp-3*, *V5*::*syp-4*, *OLLAS*::*ddb-1*, *FLAG*::*gad-1*, and *cul-4*::*pole-1-3’UTR*

All CRISPR/Cas9 reactions used ssODNs and crRNAs generated by Integrated DNA Technologies (IDT), materials used are listed below in the table. Recombinant Cas9 protein was isolated by QB3 MacroLab (University of California, Berkeley). A *dpy-10* co-injection marker was used as a measure of successful Cas9 excision. CRISPR/Cas9 was performed as described in [86](with slight modifications) and the co-CRISPR protocol described in [87]. *spo-11* and *ddb-*1are linked therefore the double mutant was created by CRISPR/Cas9. Deletion of *spo-11* was generated as in [88] but by injection into *ddb-1(tm1769/nT1* strain [*spo-11(iow110)* deletion was verified as homozygous the PCR of *ddb-1(tm1769)* homozygotes, non-balanced worms].

**Table:** 

Gene target	ssODN	crRNA
*syp-1*	5’-catactgcagatgttcgccgaaagagaggagggaagaaagactacaaagaccatgacggtgattataaagatcatgaTatcgaTtacaaggatgacgatgacaagtaatgtgtgtgtggggaagaaacgactatgtaccatttcaatc -3’	5’- GGGAAGAAATAATGTGTGTG -3’
*syp-2*	5’- GTCTTAAAAcgagagccgaagctcatactgcagatgttcgGcgTaagCgaggaggCaagaaaggtaagcctatccctaaccctctcctcggtctAgatAGTacTtaatgtgtgtgtggggaagaaacgactatgtaccatttcaatcttgtgct -3’	5’- tggttgaaacCTtGgagccTtgg -3’
*syp-3*	5’- ggccatttcaatatctccaatttcagagataaATGgactacaaagaccatgacggtgattataaagatcatgaTatcgaTtacaaggatgacgatgacaagAATTTCGAAAAGCTTGTCAGTCAGGCAGTAAATGGTGACCGTTTTAAAATTTTTTGTGGGCAGCTCACTGAGTTCACCAACTCGCTCGCTGGGGAAAG -3’	5’- AATTCATTTATCTCTGAAAT -3’
*syp-4*	5’- gttcggtacggtaacctcatttttcatcaaaattttttatttcaaggcgaaataatgggtaagcctatccctaaccctctcctcggtctAgatAGTacTtcgtttccgacgTtacaagtGAgAccaaatgagaaaaatccaaaagttctgcgatgcc -3’	5’- tttggTcTCacttgtaAcgtcgg -3’
*ddb-1*	5’- tacagtaattggaatgatagtcgagacgataatcactttcccatgagacgtggtccGAGCTCgttggcgaatccggagtgcattctcgcTaaatcctcgatgactttgagaatttcaactggatctctt -3’	5’- tctcaaagtcatcgaggatt -3’
*gad-1*	5’- gacagatgaaaaacaaaaaattaatgcaacgactacttgtcatcgtcatccttgtaAtcgatAtcatgatctttataatcaccgtcatggtctttgtagtccttcgttctcggcattttAaaCacaggctgtagctcttcgtcatcttgatcttctg -3’	5’- gttctcggcattttgaagac -3’
*cul-4*	5’-aataatctaacattttcaacagaacaaattggatggactacaaagaccatgacggtgattataaagatcatgaTatcgaTtacaaggatgacgatgacaagacatctggagcaccaccgactatttcaacagaaaaaa -3’	5’-agaacaaattggatgacatc-3’
*cul-4*::pole-13’UTR	5’- aatttcgtataaagaattcaatcaaataaccatgtaaatagctgaaaatgaattcattggaaaaatgagtagatatatgttacagtaaaaagtagttacccgaaaaaatacaaccaaaaaaattagggaaatttacgcaacataattatagctagatgcTtcttcaggatccctcgatatatattcacgttcgatgagt -3’	5’- ccaaaatactgttgaataca -3’

### RNAi Screen

To identify possible adapter proteins and E2 conjugating enzyme(s) involved in SC assembly, we performed RNAi knockdown of 41 different possible adapter proteins, containing the DDB1 binding WD40 domain (DWD), and 22 predicted E2 enzymes. The screen was performed in an *akir-1(gk528);rrf-1(pk1417)* double mutant background. Previous work in our lab has shown that *akir-1(gk528)* mutants can be used as a sensitized background to find genes that are involved in SC assembly [34,50]. *rrf-1* is a gene encoding an RNA dependent RNA polymerase expressed in somatic tissue; *rrf-1* mutants were used to direct RNAi knockdown specifically to the germline, as these RNAi targets likely have a function in somatic tissue. A *gfp*::*syp-3* transgenic line was crossed into these double mutants to quantitatively determine if each knockdown condition influenced SC assembly. Germlines were examined for PC formation in pachytene; PCs were defined as SC structures that are at least two times the width of linearized SC. In this screen, we exposed our double mutants with the *gfp*::*syp-3* transgenic line to RNAi by feeding and performed whole worm ethanol fixation for analysis. RNAi clones were inoculated overnight in Lauria broth and ampicillin (50 ng/μl). Bacterial cultures were seeded on plates containing IPTG then allowed to grow for a minimum of 12 hrs. at 37°C. RNAi clones from the Ahringer *C*. *elegans* RNAi library were used [89]. L1 synchronized larvae were placed on the seeded RNAi bacterial plates, *pL4440* empty vector was used as a control. Once the next generation reached L4 stage, they were moved to another set of RNAi seeded plates. The next generation at L4 stage were further moved to another set of plates and as day 1 adults were ethanol fixed and examined for PC formation.

### Immunofluorescent staining and microscopy

Adult hermaphrodites were dissected, genotypes were dissected at different times, based on growth rate, after L4 stage. Wild type, *csn-5(ok1064)*, *cand-1(tm1683)*, *rbx-2(ok1617)*, *spo-11(ok79)*, *rad-54(ok615)*, *msh-5(me23)*, and *rad-51(ok2218)* strains were dissected 20–24 hrs. post-L4. *ddb-1(tm1769)*, *gad-1(ok573)*, and *rbx-1(ok782)* strains were dissected 30–35 hrs. post-L4. The *cul-4(ok1891)* strain was dissected 48–52 hrs. post-L4. Immunofluorescent staining antibodies used are as follows: α-SYP-1 (1:500, VDAPTEALIETPVDDQSSGFL), α-RAD-51 (1:10,000; ASRQKKSDQEQRAA), α-HTP-3 (1:500; gift from M. Zetka), α-HIM-8 (1:1,000; #4198.00.02, Sdix), α-FLAG (1:500; #F1804, Sigma), and α-OLLAS (1:500; #A01658, Genscript). Secondary antibodies used were α-goat Alexa Fluor 549, and α-rabbit Alexa Fluor 488. All gonads were stained with DAPI (1:2,000 dilution of a 5-mg/ml DAPI stock in PBS-Tween) for 10 min. Immunostaining was performed as in [22]. In short, worms were dissected in M9 buffer and freeze cracking was applied. Dissected germlines were then incubated in methanol, followed by 4% PFA of 30 minutes at room temperature. 1X PBS-Tween was used as wash buffer. Fixed samples were imaged with Vectashield medium. DeltaVision wide-field fluorescence microscope system (Applied Precision Ltd.) with Olympus 100x/1.40-NA lens was used for image acquisition. Using a coolSNAPHQ camera (Photometrics) and softWoRx software version 7.0.0. (Applied Precision Ltd.), 0.20-μm optical sections were collected. Images were deconvolved using softWoRx. Adobe Photoshop CC was used for image processing. Images were adjusted after assembly using the levels function; images of wild type and mutants in the same panel were manipulated identically and simultaneously.

### Sample size for figures

GraphPad Prism was used for generating of all graphs and statistical analyses. *n* reflects the number of either germlines or nuclei analyzed. **Fig 1:** C- 10 germlines for all genotypes analyzed; D- 1,712 nuclei; E- 695 nuclei; F- 755 nuclei; G- 657 nuclei; H- 619 nuclei; I- 711 nuclei; J- 1,194 nuclei; K- 1,613; from 3 independent germlines for each genotype. K- wild type 28, *csn-5(ok1064)* 39, *cul-4(ok1891)* 39, *ddb-1(tm1769)* 29, *gad-1(ok573)* 23, *rbx-1(ok782)* 27, *rbx-2(ok1617)* 35, *rbx-1(ok782);rbx-2(ok1617)* 25, *cand-1(tm1683)* 21 germlines analyzed. L wild type 189 *cul-4(ok1891)* 162 SC and 47 PC nuclei, **Fig 2: Fig 3:** A- wild type 50, *flag*::*cul-4* 70, *flag*::*cul-4(ok1891)* 90, and *flag*::*cul-4*::*pole-1 3’UTR* 70, B- From left to right: wild type 24, 38, 12, 16,13 and 8 gonads, *flag*::*cul-4*::*pole-1 3’UTR* 27, 14, 12, 8, 28 and 14 gonads. C- wild type 711 nuclei, *cul-4(ok1891)* 300 nuclei, D- wild type 15, 24 and 9 germlines, *cul-4(ok1891)* 15, 39 and 20 germlines, *cul-4(ok1891)/+* 21 and 23 germlines, *ddb-1(tm1769)* 34 and 29 germlines, *gad-1(ok573)* 5 and 23 germlines, **Fig 3:** B- wild type 866 nuclei, *csn-5(ok1064)* 592 nuclei, *cul-4(ok1891)* 436 nuclei, *ddb-1(tm1769)* 502 nuclei; D- wild type 907 nuclei, *csn-5(ok1064)* 625 nuclei, *cul-4(ok1891)* 532 nuclei, *ddb-1(tm1769)* 346 nuclei from 3 independent germlines for each genotype. **Fig 4:** A- 1,124 nuclei; B- 552 nuclei; C- 665 nuclei; D- 627 nuclei; E- 1,396 nuclei; F- 755 nuclei; G- wild type 1,124 nuclei, *csn-5(ok1064)* 552 nuclei, *cul-4(ok1891)* 665 nuclei, *ddb-1(tm1769)* 515 nuclei, and *rad-54(ok615)* 812 nuclei from 3 independent germlines G 100 nuclei, H- 100 nuclei, I- 10 nuclei per genotype. **Fig 5:** A- 1,124 nuclei; B- 665 nuclei; C- 1,133 nuclei; D- 422 nuclei; E- 1,444 nuclei; F- 560 nuclei; G- 1,701 nuclei; H- 431 nuclei from 3 independent germlines; I- Between 30 and 70 worms were used for each sample, 4 biological replicates with 3 technical replicates were analyzed for each genotype. **Fig 6:** B- wild type 39, *csn-5(ok1064)* 60, *cul-4(ok1891)* 83, *ddb-1(tm1769)* 43, *gad-1(ok573)* 55, *cand-1(tm1683)* 89, *rbx-1(ok782)* 79, *rbx-2(ok1617)* 79 nuclei were analyzed from 3 independent germlines; D- wild type 22, *rbx-1(ok782)* 20, *rbx-2(ok1617)* 21, *cand-1(tm1683)* 20 diakinesis-1 nuclei were analyzed. **Fig 7:** A- 1,712 nuclei; B- 657 nuclei; C- 1,694 nuclei; D- 439 nuclei; E- 1,629 nuclei; F- 501 nuclei; G- 1,646 nuclei; H- 701 nuclei; I- 1,399 nuclei; J- 610 nuclei; K- wild type 28, *cul-4(ok1891)* 39, *spo-11(ok79)* 22, *cul-4(ok1891);spo-11(ok79)* 23, *rad-51(ok2218)* 23, *cul-4(ok1891);rad-51(ok2218)* 28, *rad-54(ok615)* 20, *cul-4(ok1891);rad-54(ok615)* 33; *msh-5(me23)* 27; *cul-4(ok1891);msh-5(me23)* 30, *csn-5(ok1064)* 39, *csn-5(ok1064); spo-11(ok79)* 31, *ddb-1(tm1769)* 29, *ddb-1(tm1769); spo-11(ok79)* 19, *cul-4*::*pole-1-3'UTR; spo-11(ok79)/nT1* 18 23°C, *cul-4*::*pole-1-3'UTR; spo-11(ok79)* 28 23°C, *cul-4*::*pole-1-3'UTR; spo-11(ok79)/nT* 11 25°C, *cul-4*::*pole-1-3'UTR; spo-11(ok79)* 10 25°C, germlines analyzed. **S4 Fig:** B through E- at least 3 replicates of each western was performed with whole worm lysate from 30–50 worms per genotype, **S4 Fig:** wild type 12, *cul-4(ok1891)* 12 DHC-1 patches; from at least 3 independent germlines per genotype, **S6 Fig:** A- 627 nuclei; B- 1,328 nuclei; C- 1.592 nuclei; D- 1.570 nuclei; E- 1,493 nuclei; F- 507 nuclei; from 3 independent germlines per genotype.

### Germline length microscopy and analysis

Adult hermaphrodites were fixed with ethanol and sealed with DAPI and Vectashield upon reaching day one adult stage. Full worm images were taken with a Leica DM RXA compound light microscope with QI Click Camera 20X lens. Images were analyzed using softWoRx software by calculating the distance between the distal tip cell to the end of pachytene. Ten worms were analyzed per genotype. Statistical comparison: two-tailed Mann-Whitney.

### Germline zonal determination

Germlines were divided into zones based on length: wild type length having seven zones and *cul-4(ok1891)* mutants having six zones. Each zone represented a time point in meiotic prophase I progression (described in Results; *SC assembly is perturbed in CRL4 mutants*). Zones were created by imaging from the distal tip cell using a 512x512 pixel dimension frame of view. Images were taken until the end of pachytene.

### GFP::SYP-3 analysis

Adult hermaphrodites were fixed with ethanol and sealed with DAPI and Vectashield upon reaching day one adult stage. Whole worms were observed under the Deltavision System described earlier. Germlines were examined for GFP::SYP-3 PC formation (see *RNAi Screen* for PC definition).

### FISH

5S locus FISH and analysis was performed as in [21]. Creation of the 5S rDNA FISH probe was done by amplification of the 1kb 5S rDNA locus with primers: 5′-TAC TTG GAT CGG AGA CGG CC-3′ and 5′-CTA ACT GGA CTC AAC GTT GC-3′ and fluorescent labeling with terminal deoxynucleotidyl transferase. Freeze crack method was used as in *Immunofluorescent Staining and Microscopy* except with 7.4% PFA applied before freezing. 2X SSC-tween was used as buffer. Hybridization of the 5S rDNA probe to germline nuclei was performed at 94°C for 90s. Three germlines per genotype were scored.

### FISH and SYP-1 immunostaining

Germline squash preparations were prepared as in [85]. Squash preparations were first processed as in “Immunofluorescent Staining and Microscopy” with α-SYP-1 (1:500, VDAPTEALIETPVDDQSSGFL) and secondary antibody α-goat Alexa Fluor 549. Nuclei were fixed in 3.7% PFA then hybridized with the 5S rDNA probe as in “FISH”. Nuclei were then stained with DAPI. Analysis of homologous synapsis in pachytene nuclei was performed by searching for nuclei with linear SYP-1 and at least one 5S rDNA focus residing on or next to linearized SYP-1. Paired foci were determined by a distance of 0.7μm or less between 5S rDNA foci; as in “FISH and HIM-8 Analysis” below.

### Western blot

Whole worm lysis was flash frozen in SDS urea lysis buffer and 2-mercaptoethanol (S3A, S3B & S3E Fig), or whole worm lysis was frozen and incubated with SDS urea lysis buffer and 2-mercaptoethanol during thaw (S3C, S3D & S3F Fig). Samples were boiled and run on a 10% SDS Express plus PAGE gel (#M01012; GenScript). 1X PBS-Tween was used as wash buffer. Antibodies used were as follows: α-V5 (1:2,000- S3A, S3B & S3E Fig, #46–0705; 1:5,000- S3C, S3E & S3F Fig, R960-25; Invitrogen), α-FLAG (1:3,000; #F1804, Sigma-Aldrich), and α-tubulin (1:1,000- S3A, S3B & S3E Fig; 1:10,000- S3C, S3D & S3F Fig; ab1157911, Developmental Studies Hybridoma Bank) used as loading control. Secondary antibodies used were α-mouse antibody conjugated to HRP (1:10,000- Sup 3A, B, & E; 1:5,000- Sup 3C, D, & F). 5% milk in 1X PBS-Tween was used for blocking (S3A, S3B & S3E Fig; no blocking was used in S3C, S3D & S3F Fig). WesternBright ECL (#K-12045-D20; Advansta) was used for HRP detection and blot development. The LI-COR Odyssey Infrared Imaging System was used for development and analysis of western blots. For proteasome inhibition 100 adult worms were incubated in 1μM MG132 (EMD Millipore) for 8 hours, 20-24hrs after L4 stage selection. These worm incubations were then used for western blot analysis.

### RT-PCR

RT-PCR was performed in *cul-4(ok1891)* strains. Superscript III OneStep RT-PCR kit (12574–026; Thermo Fisher Scientific) and primers 5’-ttagctgctttcgagccttc-3’ and 5’-gcttcaaatccgcaaatgat-3’. The obtained RT-PCR fragment was sequenced to reveal a deletion of part of exon 10 and most of exon 11 which created an out of frame deletion (junction sequence: TCTCGATCAAATGGTA/AGAAGGAAGGTACTGTG).

### qRT-PCR

Quantitative RT-PCR was performed on *Tc1* and *Tc3*, *TC1/Mariner* class DNA transposable elements, in wild type and *cul-4(ok1891)* strains. Superscript III OneStep RT-PCR kit (12574–026; Thermo Fisher Scientific) and primers designed as in [13] were used. *Tc1*: 5’-CTT GAA GCG CTT CTT GTC ACGC-3’ and 5’-CCA ACC ACT GGA ACG ACC GTG-3’; *Tc3*: 5’-GAG CGT TCA CGG AGA AGA AG-3’ and 5’-AAT AGT CGC GGG TTG AGT TG-3’. The Roche LightCycler 480 Real-Time PCR System was used for qRT-PCR and analysis of *Tc1* and *Tc3* transcript abundance. Actin RT-PCR was used for normalization: 5’-ATC ACC GCT CTT GCC CCA TC-3’ and 5’-GGC CGG ACT CGT CGT ATT CTT-3’. 30 worms with equal numbers of wild type and *cul-4(ok1891)* mutants used in each qRT-PCR prep. Each qRT-PCR prep was split into 3 technical controls. For each genotype, 3–4 biological controls were used. Samples were excluded if Cts > 29 for actin or if Ct = 40 for Tc3 in any of the replicas. Each Tc3 sample was normalized to actin and two paired samples were subtracted to get ΔΔCT. Relative expression was obtained from ΔΔCT.

### SYP-1 localization analysis

Qualitative scoring of SYP-1 localization patterning within individual nuclei was determined by placement into one of eight different categories. “No SYP-1” was defined as having no SYP-1 immunofluorescence present along chromosomes (DAPI). “SYP-1 PC” were nuclei with only PC formation present, no elongation of SYP-1 along DAPI. “SYP-1 PC and Some Linear” was the presence of PC(s) in the nucleus but also partial elongation of SYP-1, <50% of DAPI. “SYP-1 PC and Linear” was similar to “SYP-1 PC and Some Linear” with the exception that SYP-1 is elongated along >50% of DAPI. “SYP-1 Partial Linear” nuclei had elongated SYP-1 along up to 50% of DAPI. “SYP-1 Mostly Linear” nuclei had SYP-1 along up to 50% of DAPI but less than 100%. “SYP-1 Linear” nuclei had fully elongated SYP-1 along all DAPI. “Other” was defined as nuclei that had abnormal DAPI appearance, for example in *csn-5(ok1064)* mutants this represented micronuclei formation, and in *cul-4(ok1891)* mutants this represented an EMO-like dispersed DAPI appearance. Statistical comparison: Fisher’s exact test.

### RAD-51 foci analysis

All zones were quantified for number of RAD-51 foci per nucleus as in [22]. Three germlines were scored per genotype and the mean was determined. Statistical comparison: two-tailed Mann-Whitney.

### Fluorescence intensity measurements

16-bit non-deconvolved images acquired with a one second exposure were analyzed by FIJI software. Intensity measurements using anti-FLAG antibody were gathered by drawing a circle around a nucleus at its greatest width from a single Z-stack from PMT or LP. For each genotype and region, 10 nuclei per germline were measured for three or more germlines. Background was recorded at 4 different positions inside the cytoplasmic space and the average of the cytoplasmic intensities was subtracted from each nuclear intensity recording. Mann-Whitney U test (GraphPad Prism 7 software) was used to statistically compare intensities of staining. For RAD-51 intensity analyses, images acquired for *RAD-51 Foci Analysis* were used. Full nuclear projections were created and analyzed in ImageJ. An ellipse (1 pixel wide) was placed around a targeted nucleus, based on DAPI staining, using the circle tool. Mean intensity of the RAD-51 channel was calculated and divided by the number of RAD-51 foci with that nucleus. These values were then normalized to the mean intensity of the RAD-51 channel in an ellipse placed in the cytoplasm. Statistical comparison: two-tailed Mann-Whitney.

### Irregular RAD-51 foci analysis

Images acquired for *RAD-51 Foci Analysis* were used. Individual nuclei were analyzed for abnormal RAD-51 appearances. Abnormal RAD-51 were defined as anything that did not form wild type puncta. Examples of this include stretches of RAD-51 or a globular “lobed” appearance larger than wild type puncta. Statistical comparison: Fisher’s exact test.

### 1,6-Hexanediol treatment and analysis

Adult worms were dissected in Egg buffer as in timeline for different mutant strains described in *Immunofluorescent Staining and Microscopy* after L4 stage selection. Dissected germlines were incubated in 15% 1,6-Hexanediol then fixed in 3.7% PFA. Germlines were then immunofluorescent antibody stained for SYP-1 and HTP-3. Using the DeltaVision system and softWoRx software, images were analyzed for presence of SYP-1 PC formation and linearization along the axial element HTP-3. Statistical comparison: Fisher’s exact test.

### FISH and HIM-8 analysis

Zonal division, as in SYP-1 *Localization Analysis*, was used to divide germlines and were scored for 5S rDNA FISH signal/HIM-8 foci pairing in each nucleus. Paired foci were determined as being 0.7-μm or less apart [21]. Statistical comparison: Fisher’s exact test. Simultaneous antibody staining and FISH was performed on squashes as in [17].

### COSA-1 foci analysis

GFP::COSA-1 was quantified in the final zone of each germline (either Zone 6 or 7 dependent upon germline length), corresponding to late pachytene [59]. COSA-1 foci were counted per nucleus with a scoring of at least three germlines per genotype. Statistical comparison: two-tailed Mann-Whitney.

### Diakinesis nuclei analysis

Images were taken of the final diakinesis nucleus (diakinesis -1) prior to spermathecal entrance. Number of DAPI bodies were totaled following counting in 3D. Statistical comparison: two-tailed Mann-Whitney.

### DHC-1::GFP movement analysis

Analysis of movement was performed in DHC-1::GFP transgenic strains in the wild type (N2) and *cul-4(ok1891)* mutant background. Images of single plane at TZ (based on distance from tip and presence of DHC-1::GFP foci) were taken for 60 s. Analysis was performed in ImageJ using the plugin Manual Tracking for tracking of DHC-1::GFP foci. Data points were taken at the onset and conclusion of each directional movement, obtaining the calculation of total distance traveled, mean velocity, number of times foci changed direction, and time between movements of foci. All DHC-1::GFP foci visualized during the whole duration of the movie (60s) were analyzed. Statistical comparison: two-tailed Mann-Whitney.

### Statement on data and reagent availability

Strains are available upon request.

## Supporting information

S1 Fig CRL4 E3 ligase complex mutants exhibit PC formation during SC assembly. A, B, & D-J) Representative images of SYP-1 immunofluorescent staining in CRL4 mutants throughout progression of the germline. Blue (DAPI) and red (SYP-1). Scale bars are 2μm. C) Table representing analysis of GFP::SYP-3 in the *cul-4(ok1891)* mutant background. Results are grouped into three categories: whole worms with no PC formation in either germline, worms with PC formation in one germline, or worms with PC formation in both germlines. The number of germlines with PCs is not significantly different from observations in SYP-1 immunofluorescent analyses. Scale bars are 2μm. K-N) Immunostaining using antibodies against HTP-3 (green) and SYP-1 (red) in CRL mutants shows that HTP-3 localized to chromosomes in linear fashion about the ame time SYP-1 appears, in all genotypes analyzed. Scale bars are 5μm.
(TIF)

S2 Fig GAD-1, CUL-4, and DDB-1 localize to meiotic germline nuclei. A) Representative images of immunofluorescent staining against OLLAS is presented as progression through meiotic prophase I of the *ddb-1*::*OLLAS* transgenic line. B) Representative images of immunofluorescent staining against FLAG is presented as progression through meiotic prophase I of the e *gad-1*::*FLAG* transgenic line. C) Representative images of immunofluorescent staining against FLAG in late pachytene of the *FLAG*::*cul-4* transgenic line. Circles indicate nuclei, as an example of the quantification in Fig 2A. Top (A and B)/left (C): Blue (DAPI) and green (FLAG/OLLAS), bottom (A and B)/right (C): grey (OLLAS/FLAG). All lines were generated through CRISPR/Cas9 insertion. Scale bars are 2μm.
(TIF)

S3 Fig No evidence for SYP ubiquitination in CRL4 mutants. A-F) Western blot analysis of SYP proteins (*syp-1*::*FLAG*; *syp-2*::*V5*; *FLAG*::*syp-3*; *V5*::*syp-4*) was performed with whole worm lysates. Expected sizes for SYPs are: SYP-1 56.6 kDa, SYP-2 23.7 kDa, SYP-3 25.8 kDa and SYP-4 67.3 kDa. See Materials and Methods for antibodies and dilutions. G-J) Western blot analysis of SYP proteins as in A-F but with proteasome inhibition (MG132). In J a shift was observed for SYP-3 in *cul-4(ok1891)* mutant background but this was not repeated in 2 other blots. For G and I the same wild type control is used. K is quantification of standard western blots, while L is quantification with proteasome inhibition. For K and L all replications were included (n of at least 3 western blots).
(TIF)

S4 Fig CRL4 E3 ligase complex mutants that show meiotic defects also exhibit persistent SUN-1 patches. A-I) Representative images of SUN-1 immunofluorescent staining in CRL4 mutants in TZ and LP, genotypes indicated on the side. Blue (DAPI) and green (SUN-1). SUN-1 patches are present in all genotypes at TZ, but some LP nuclei contain patches as well in CRL4 mutants that form PCs or show accumulation of recombination intermediates (C, G, B and H). Scale bars are 2μm. J-M) analysis of movement of DHC-1::GFP foci in wild type and *cul-4(ok1891)* mutants in TZ nuclei shows no requirement for CUL-4 in chromosome movement.
(TIF)

S5 Fig CRL4 E3 ligase complex mutants have increased levels of meiotic recombination intermediates (RAD-51). A-D) Left: representative images of RAD-51 immunofluorescent staining in CRL4 E3 ligase mutants. Blue (DAPI) and green (RAD-51). Right: graphical analyses of RAD-51 foci appearance throughout the germline. Statistical comparisons were compared to wild type worms (Mann Whitney; p-values, * < 0.05). E & F) Left: representative images of RAD-51 immunofluorescent staining. Right: analyses of number of RAD-51 foci per nucleus throughout meiotic prophase I. Blue (DAPI) and green (RAD-51). Statistical comparisons of double mutants were made against single mutants (Mann-Whitney; p-values, * < 0.05). Scale bars are 2μm. G) Fold change values in *cul-4(ok1891)* and *ddb-1(tm1769))* mutants (Tc3 expression normalized to actin) average +/- SEM.
(TIF)

S6 Fig A model for CRL4 function in SC assembly. A) SC assembly in the genotype tested, B) the connection between recombination and PC formation in CRL mutants. The structure of the CUL4 complex is based on work in other organisms. Physical interaction between DDB-1 and CUL-4 was shown in *C*. *elegans* by others [40].
(TIF)

S1 File Underlying numerical data. This file contains the underlying numerical data for all the figures. Each tab corresponds to a figure panel with numerical data.
(XLSX)
