# Supplementary figures and images for: CRL4 regulates recombination and synaptonemal complex aggregation in the Caenorhabditis elegans germline
Source: PLoS Genet. 2019 Nov 18;15(11):e1008486. doi: 10.1371/journal.pgen.1008486 (PMC6886871; doi:10.1371/journal.pgen.1008486)

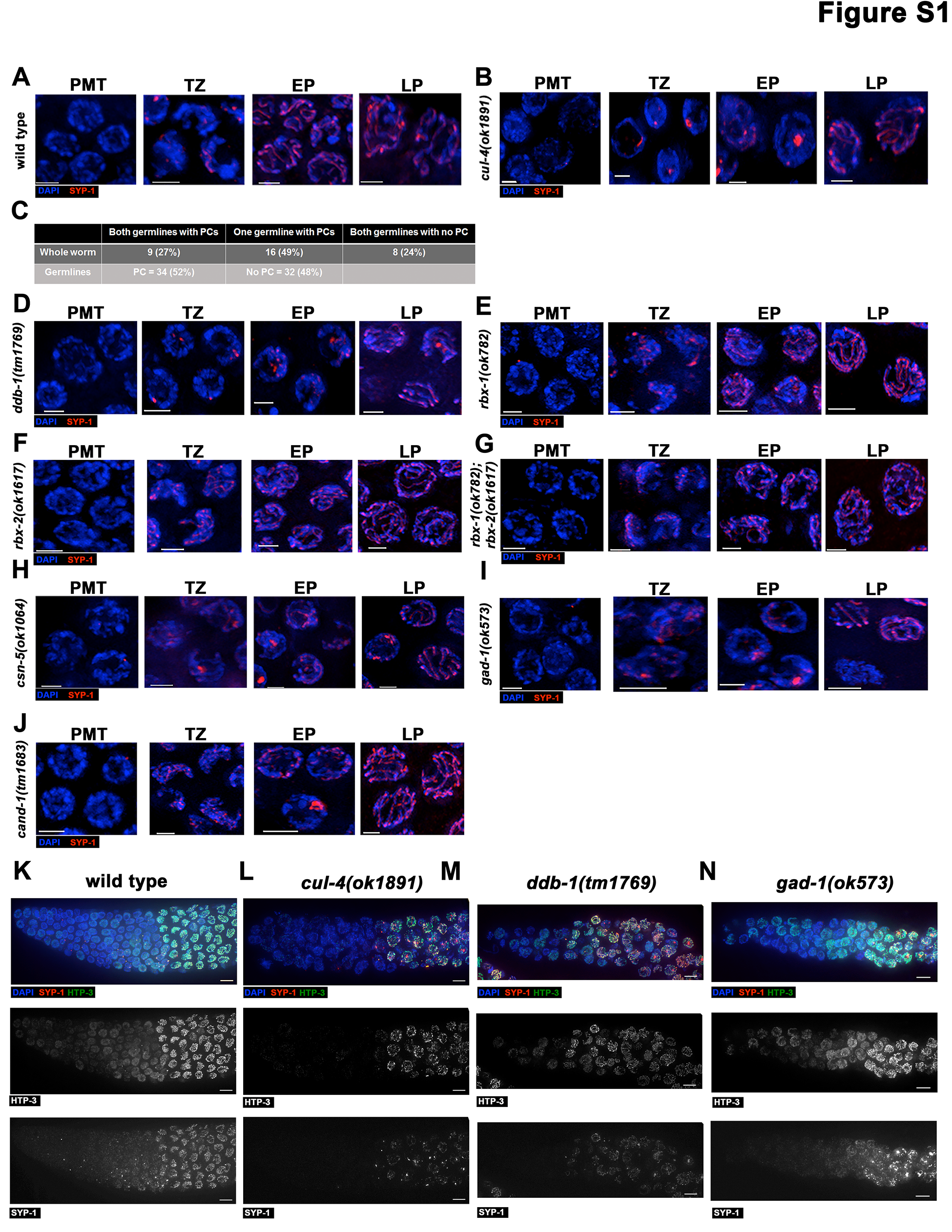

Supplement: S1 Fig — A, B, & D-J) Representative images of SYP-1 immunofluorescent staining in CRL4 mutants throughout progression of the germline. Blue (DAPI) and red (SYP-1). Scale bars are 2μm. C) Table representing analysis of GFP::SYP-3 in the cul-4(ok1891) mutant background. Results are grouped into three categories: whole worms with no PC formation in either germline, worms with PC formation in one germline, or worms with PC formation in both germlines. The number of germlines with PCs is not significantly different from observations in SYP-1 immunofluorescent analyses. Scale bars are 2μm. K-N) Immunostaining using antibodies against HTP-3 (green) and SYP-1 (red) in CRL mutants shows that HTP-3 localized to chromosomes in linear fashion about the ame time SYP-1 appears, in all genotypes analyzed. Scale bars are 5μm. (TIF) [file pgen.1008486.s001.tif]

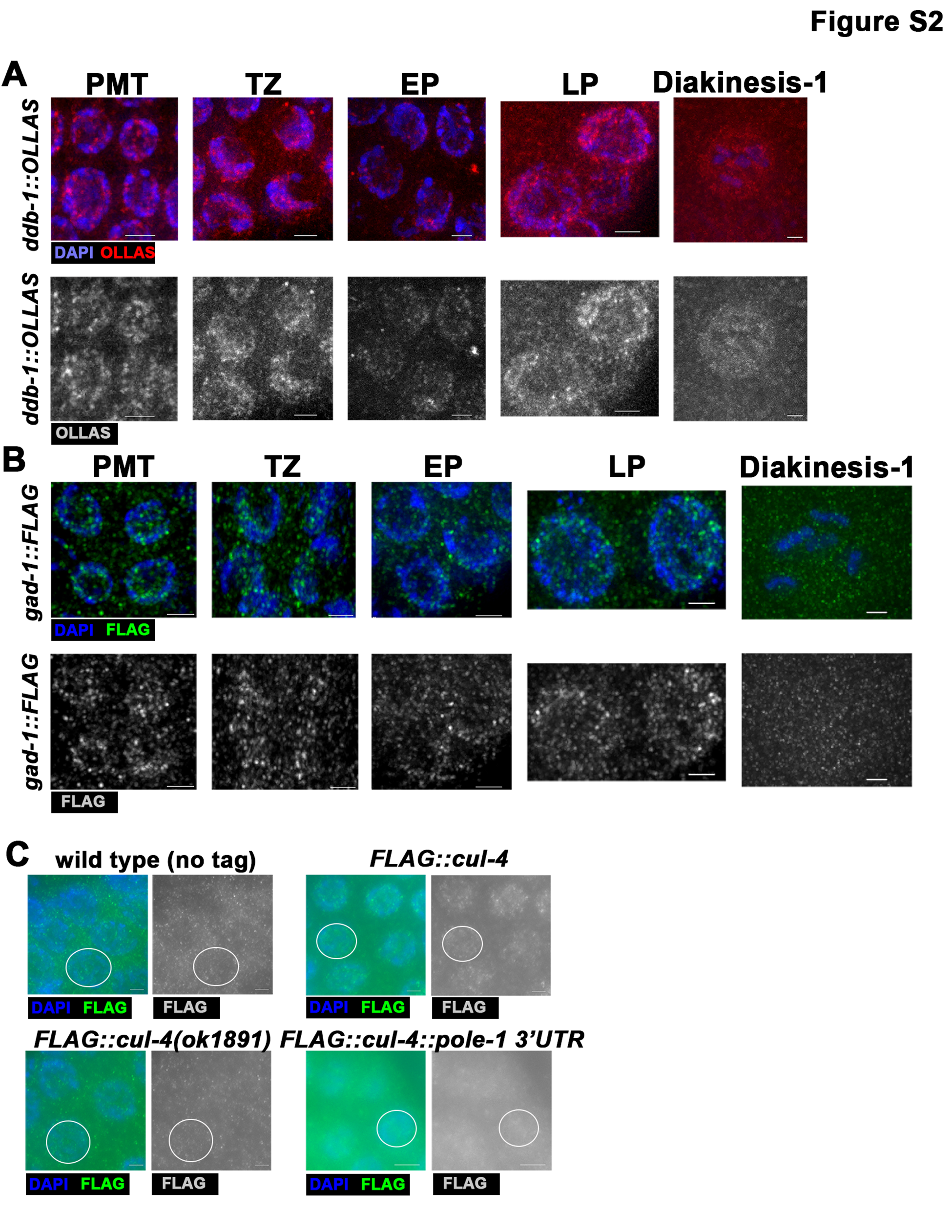

Supplement: S2 Fig — A) Representative images of immunofluorescent staining against OLLAS is presented as progression through meiotic prophase I of the ddb-1::OLLAS transgenic line. B) Representative images of immunofluorescent staining against FLAG is presented as progression through meiotic prophase I of the e gad-1::FLAG transgenic line. C) Representative images of immunofluorescent staining against FLAG in late pachytene of the FLAG::cul-4 transgenic line. Circles indicate nuclei, as an example of the quantification in Fig 2A. Top (A and B)/left (C): Blue (DAPI) and green (FLAG/OLLAS), bottom (A and B)/right (C): grey (OLLAS/FLAG). All lines were generated through CRISPR/Cas9 insertion. Scale bars are 2μm. (TIF) [file pgen.1008486.s002.tif]

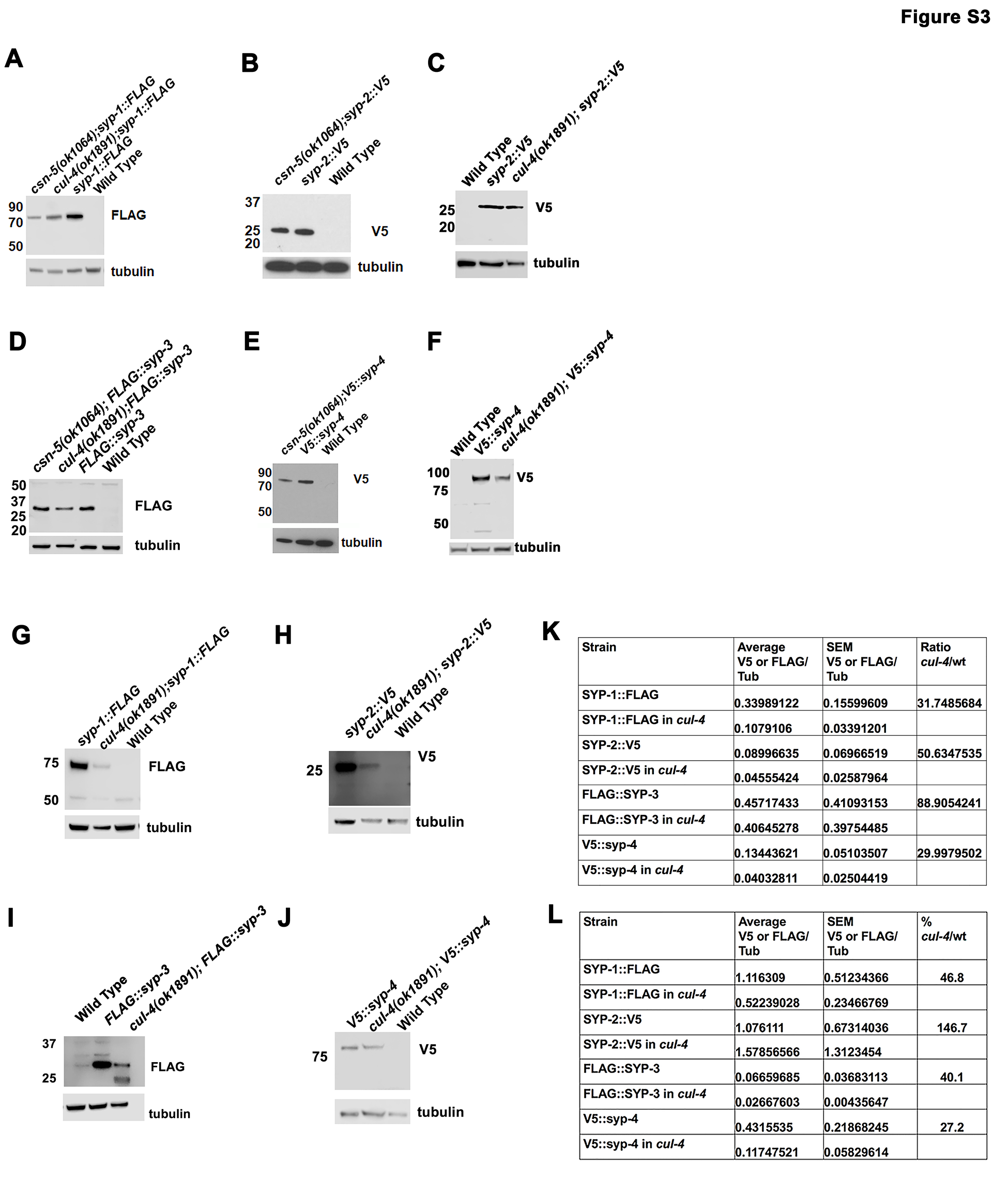

Supplement: S3 Fig — A-F) Western blot analysis of SYP proteins (syp-1::FLAG; syp-2::V5; FLAG::syp-3; V5::syp-4) was performed with whole worm lysates. Expected sizes for SYPs are: SYP-1 56.6 kDa, SYP-2 23.7 kDa, SYP-3 25.8 kDa and SYP-4 67.3 kDa. See Materials and Methods for antibodies and dilutions. G-J) Western blot analysis of SYP proteins as in A-F but with proteasome inhibition (MG132). In J a shift was observed for SYP-3 in cul-4(ok1891) mutant background but this was not repeated in 2 other blots. For G and I the same wild type control is used. K is quantification of standard western blots, while L is quantification with proteasome inhibition. For K and L all replications were included (n of at least 3 western blots). (TIF) [file pgen.1008486.s003.tif]

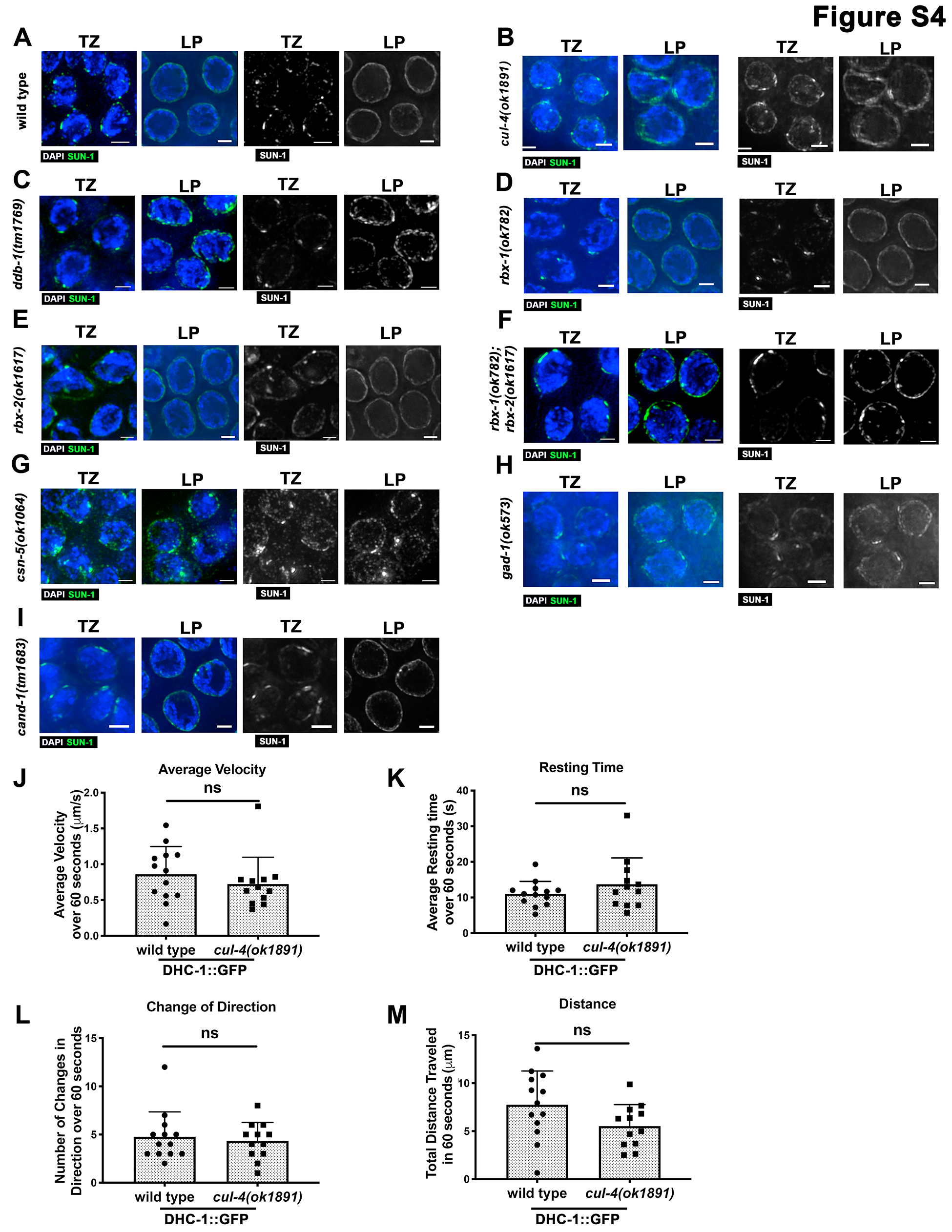

Supplement: S4 Fig — A-I) Representative images of SUN-1 immunofluorescent staining in CRL4 mutants in TZ and LP, genotypes indicated on the side. Blue (DAPI) and green (SUN-1). SUN-1 patches are present in all genotypes at TZ, but some LP nuclei contain patches as well in CRL4 mutants that form PCs or show accumulation of recombination intermediates (C, G, B and H). Scale bars are 2μm. J-M) analysis of movement of DHC-1::GFP foci in wild type and cul-4(ok1891) mutants in TZ nuclei shows no requirement for CUL-4 in chromosome movement. (TIF) [file pgen.1008486.s004.tif]

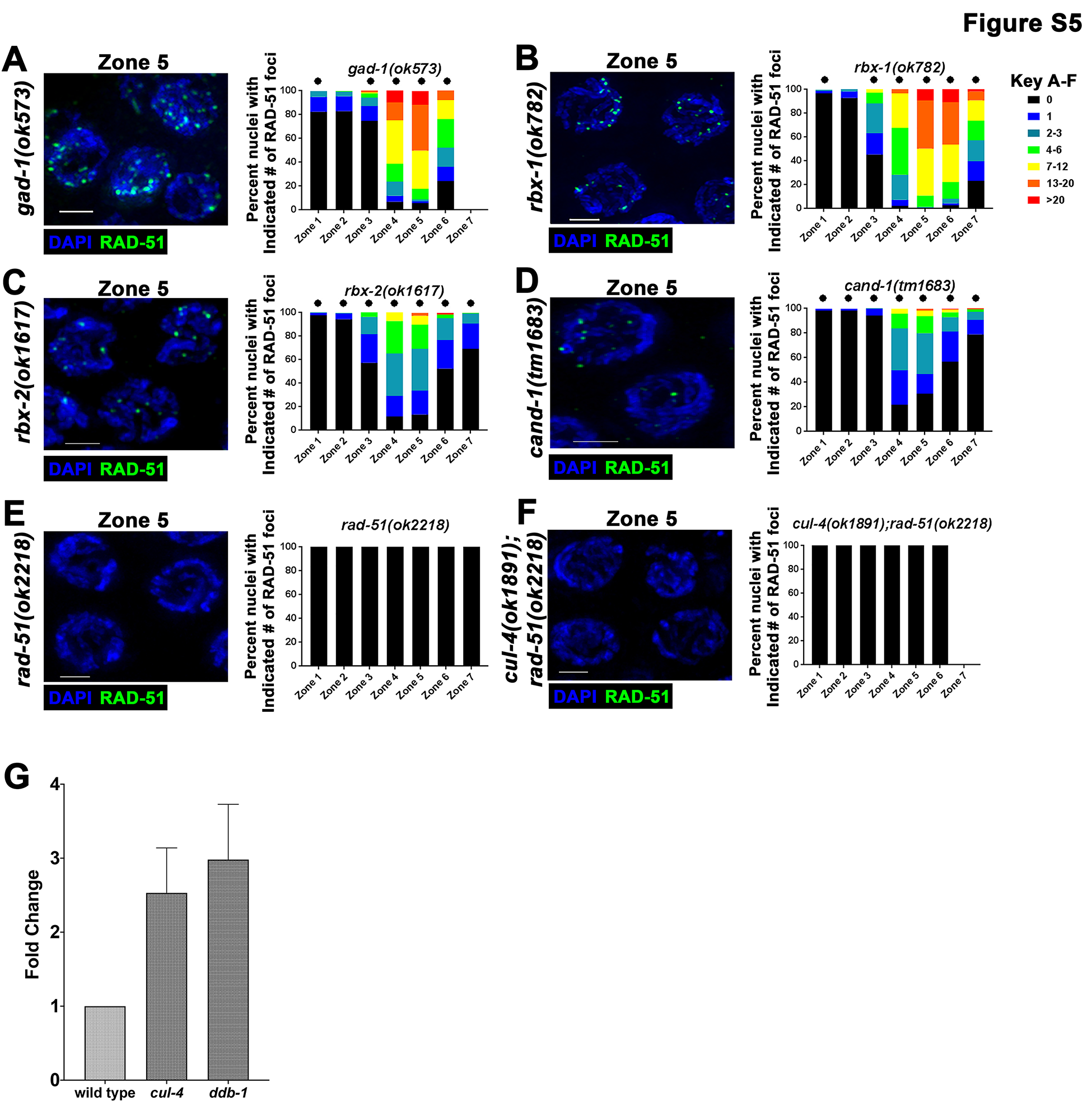

Supplement: S5 Fig — A-D) Left: representative images of RAD-51 immunofluorescent staining in CRL4 E3 ligase mutants. Blue (DAPI) and green (RAD-51). Right: graphical analyses of RAD-51 foci appearance throughout the germline. Statistical comparisons were compared to wild type worms (Mann Whitney; p-values, * < 0.05). E & F) Left: representative images of RAD-51 immunofluorescent staining. Right: analyses of number of RAD-51 foci per nucleus throughout meiotic prophase I. Blue (DAPI) and green (RAD-51). Statistical comparisons of double mutants were made against single mutants (Mann-Whitney; p-values, * < 0.05). Scale bars are 2μm. G) Fold change values in cul-4(ok1891) and ddb-1(tm1769)) mutants (Tc3 expression normalized to actin) average +/- SEM. (TIF) [file pgen.1008486.s005.tif]

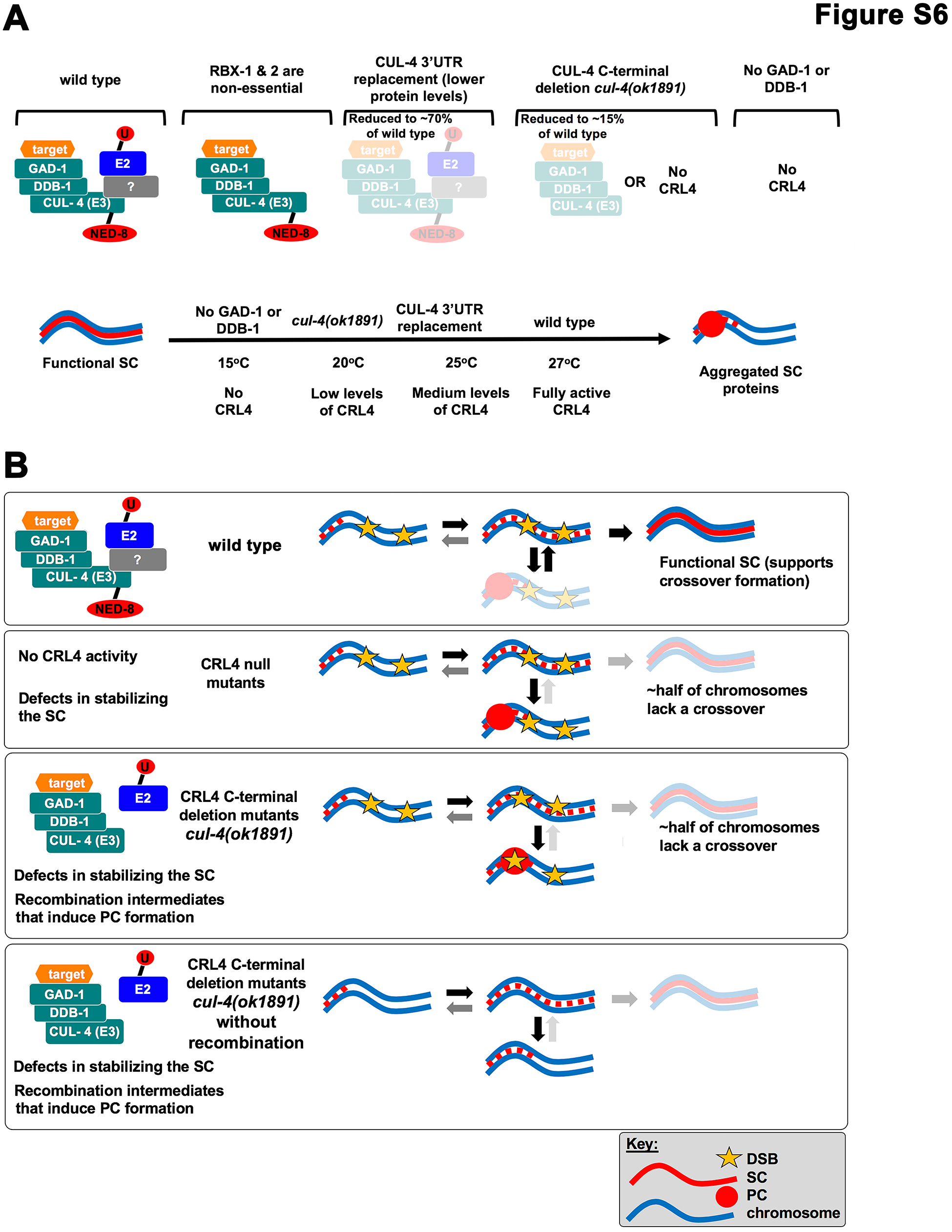

Supplement: S6 Fig — A) SC assembly in the genotype tested, B) the connection between recombination and PC formation in CRL mutants. The structure of the CUL4 complex is based on work in other organisms. Physical interaction between DDB-1 and CUL-4 was shown in C. elegans by others [40]. (TIF) [file pgen.1008486.s006.tif]
